# 7-Ketocholesterol Links Sterol Homeostasis to Hedgehog Signaling and Stress–Survival Responses in MSCs from Patients with Acute Myeloid Leukemia

**DOI:** 10.3390/ijms27062842

**Published:** 2026-03-20

**Authors:** Cadiele Oliana Reichert, Débora Levy, Fábio Alessandro de Freitas, Juliana Sampaio Silva, Priscila de Lima Barros, Jéssica Liliane Paz, João Paulo Silva Nunes, Edécio Cunha-Neto, Jorge Kalil, Pedro Nogueira Giglio, Marco Kawamura Demange, Hebert Fabricio Culler, Luís Alberto de Pádua Covas Lage, Alessandro Rodrigues, Juliana Pereira, Sérgio Paulo Bydlowski

**Affiliations:** 1 Lipids, Oxidation and Cell Biology Team, Laboratory of Immunology (LIM19), Heart Institute (InCor), Faculdade de Medicina, Universidade de Sao Paulo-HCFMUSP, Sao Paulo 05403-010, Brazil; cadiele@usp.br (C.O.R.); d.levy@hc.fm.usp.br (D.L.); fabio.alessandro@alumni.usp.br (F.A.d.F.); jukisbio@gmail.com (J.S.S.); priscila.barros@usp.br (P.d.L.B.); paz.jl@usp.br (J.L.P.); 2 Real-World Evidence & Precision Public Health Team, Laboratory of Pathogenesis and Directed Therapy in Onco-Immuno-Hematology (LIM-31), Department of Hematology, Hemotherapy, and Cell Therapy, Faculdade de Medicina FMUSP, Universidade de Sao Paulo, Sao Paulo 05419-000, Brazil; juliana.pereira@hc.fm.usp.br; 3 Laboratory of Pathogenesis and Directed Therapy in Onco-Immuno-Hematology (LIM-31), Department of Hematology, Hemotherapy, and Cell Therapy, Faculdade de Medicina FMUSP, Universidade de Sao Paulo, Sao Paulo 05419-000, Brazil; hebert.culler@hc.fm.usp.br (H.F.C.); luis.lage@hc.fm.usp.br (L.A.d.P.C.L.); 4 Instituto de Investigação em Imunologia (INCT-iii), CNPq, Sao Paulo 05403-010, Brazil; joao.psnunes@hc.fm.usp.br (J.P.S.N.); edecunha@gmail.com (E.C.-N.); jkalil@usp.br (J.K.); 5 Laboratory of Immunology (LIM19), Heart Institute (InCor), Faculdade de Medicina, Universidade de Sao Paulo-HCFMUSP, Sao Paulo 05403-010, Brazil; 6 Instituto de Ortopedia e Traumatologia, Hospital das Clinicas, Faculdade de Medicina, Universidade de Sao Paulo-HCFMUSP, Sao Paulo 05403-010, Brazil; pedrongiglio@gmail.com (P.N.G.); demange@usp.br (M.K.D.); 7 Department of Earth and Exact Sciences, Universidade Federal de Sao Paulo, Diadema 09972-270, Brazil; alessandro.rodrigues@unifesp.br

**Keywords:** 7-ketocholesterol, mesenchymal stem cells, AML, ABC transporters, liver X receptor, Sonic Hedgehog, mitochondrial bioenergetics

## Abstract

7-ketocholesterol (7-KC) is a bioactive oxysterol generated under oxidative stress and may contribute to bone marrow niche reprogramming in acute myeloid leukemia (AML), thereby promoting stress tolerance and therapeutic resistance Bone marrow mesenchymal stromal cells (MSCs) from healthy donors and AML patients were exposed to subtoxic 7-KC concentrations for 24 h. We evaluated the ABC transporters involved in lipid transport, multidrug resistance and membrane microdomain remodeling; Hedgehog pathway proteins; stress–survival signaling; redox balance by glutathione measurements, and mitochondrial function and dynamics, including membrane potential and gene expression of mitochondrial fission and fusion regulators. Results were integrated using principal component analysis (PCA), heatmaps, and correlation-based networks. Multivariate analyses revealed an integrated, lineage-dependent response. Healthy donor MSCs showed greater plasticity of the efflux and microdomain axis and higher oxidative and mitochondrial vulnerability at high 7-KC doses. AML-MSCs exhibited a basal preconditioned state phenotype and preferentially routed the response toward Hedgehog and stress–survival modules, accompanied by glutathione expansion and adaptive mitochondrial remodeling. 7-KC acts as a broad modulator of several MSC functions, linking sterol homeostasis to Hedgehog signaling, stress–survival pathways, redox balance, and mitochondrial remodeling, potentially supporting a pro-survival, more therapy-tolerant leukemic niche.

## 1. Introduction

Acute myeloid leukemia (AML) is a hematologic malignancy characterized by the clonal expansion of immature myeloid precursors and hematopoietic failure. Historically, the primary focus of research in AML has been directed toward genetic alterations and resistance mechanisms within the myeloblast [1,2]. However, over the past two decades, the bone marrow niche has gained prominence in research owing to its clear contribution to the establishment of a permissive, multidrug-resistant microenvironment that supports both disease progression and minimal residual disease [3,4,5]. The bone marrow microenvironment is complex, comprising endothelial cells, osteoblasts, immune cells, and mesenchymal stromal cells (MSCs). Several studies suggest that, under physiological conditions, MSCs exert paracrine effects and regulate hematopoietic stem cell (HSC) quiescence, self-renewal, and differentiation through the secretion of trophic factors and direct cell–cell contacts [6,7,8,9,10]. In the development of AML, the bone marrow hematopoietic niche is subverted and reprogrammed [11,12]. Leukemic cells compete for adhesion sites and growth factors, thereby inducing phenotypic changes in mesenchymal stromal cells (MSCs), which subsequently exhibit a pro-inflammatory phenotype and altered metabolic and secretory profiles [13]. These AML-altered MSCs (AML-MSCs) promote blast survival, chemoresistance, and the persistence of residual leukemic cells, thereby contributing to minimal residual disease at the expense of normal hematopoiesis [14].

Multidrug resistance (MDR) is a hallmark of AML and of the leukemia-supportive bone marrow niche, emerging from convergent mechanisms that limit intracellular drug accumulation and blunt apoptosis [15,16,17]. Among the best-characterized effectors are ATP-binding cassette (ABC) transporters, particularly ABCB1 (P-gp), ABCC1 (MRP1) and ABCG2 (BCRP), which actively extrude structurally diverse chemotherapeutics and are broadly implicated in therapy failure across cancers [18,19,20,21,22]. In addition to ABC-mediated efflux, MDR can involve non-canonical determinants, such as the lung resistance-related protein (LRP), now established as the major vault protein, the main structural component of vault particles, the large ribonucleoprotein assemblies implicated in nucleo-cytoplasmic transport and drug handling [23]. Vaults have been proposed to promote resistance by acting as intracellular drug depots, influencing drug subcellular distribution, including nuclear access, and lysosomal dynamics, thereby complementing classical efflux-based MDR pathways [24,25]. Clinically, LRP expression has been associated with chemotherapy response and outcome in AML, although the directionality of this association appears cohort- and context-dependent, underscoring the need to interrogate MDR-related networks within the specific cellular ecosystem of the leukemic niche [26,27].

Among the metabolic alterations observed in this context that are associated with multidrug resistance, the dysregulation of lipid metabolism, particularly involving cholesterol and its oxidized derivatives, stands out [28,29]. Under conditions of chronic oxidative stress and inflammation, cholesterol undergoes oxidation, giving rise to oxysterols [30]. Among these, 7-ketocholesterol (7-KC) is one of the most abundant and biologically active [31]. Frequently associated with pathologies such as atherosclerosis and neurodegeneration, 7-KC acts as a potent lipotoxic and signaling agent [31,32]. The accumulation of 7-KC triggers a cascade of deleterious intracellular and extracellular events, including the generation of reactive oxygen species (ROS), lipid peroxidation, endoplasmic reticulum stress, and mitochondrial dysfunction [33,34]. Beyond its direct toxicity, certain oxysterols such as 7-KC modulate plasma membrane architecture by altering the composition of lipid rafts and caveolae, such as caveolin-1, thereby impacting cellular sensitivity to external cues and the organization of surface receptors [33,34].

The complexity of 7-KC action lies in its nature as a bioactive oxysterol capable of modulating lipid-sensitive transcriptional pathways [35]. In particular, oxysterols can act as endogenous ligands and/or modulators of the liver X receptor (LXRα/β) axis, typically as RXR heterodimers, thereby integrating the control of cholesterol homeostasis with immune and inflammatory responses [36]. Activation of the LXR/RXR pathway promotes the transcription of efflux genes such as ABCA1 and ABCG1, which are regarded as key determinants of cholesterol efflux and reverse cholesterol transport [37,38]. In addition, pathways coordinated by PPARs, recognized as lipid sensors with broad impacts on metabolism and inflammation, may intersect with these circuits that support adaptation to lipid-induced stress [39].

In MSCs, 7-KC-driven engagement of lipid-sensitive pathways may further promote crosstalk with additional niche-relevant signaling axes, including Hedgehog signaling, which has been implicated in stromal–hematopoietic communication and stem cell maintenance and whose activation depends on Smoothened (SMO), a receptor influenced by the membrane lipid environment [40,41,42,43]. In parallel, 7-KC may intersect with pro-survival and stress response pathways like NF-κB, survivin, and BCL-2 family signaling, and with the regulation of redox balance and mitochondrial metabolism, processes that collectively support adaptation to metabolic stress [44,45,46]. Collectively, these interconnected pathways could favor cellular adaptation to metabolic stress and potentially contribute to the emergence of a leukemia-supportive, therapy-tolerant stromal phenotype [11,47]. However, how 7-KC modulates these signaling networks specifically in MSCs within the AML bone marrow niche remains insufficiently understood.

Despite the accumulated knowledge on 7-KC toxicity across diverse cellular models, the mechanisms by which this oxysterol rewires signaling networks, specifically in MSCs within the leukemic bone marrow niche, remain insufficiently defined [34,48,49,50]. Previous work from our group suggests that 7-KC cytotoxicity in AML-MSCs, and other cell lines, involves the activation of oxiapoptophagy, a cell-death program linking oxidative stress, apoptosis, and autophagy [51,52,53,54,55]. Therefore, this study aimed to characterize, in an integrated manner, the impact of subtoxic 7-KC exposure on a curated panel of pathways in healthy donor MSCs (HD-MSCs) and AML-MSCs. We profiled oxysterol processing, including nuclear receptor signaling and lipid transporters, niche-associated signaling, Hedgehog components, survival–stress axes, and redox/mitochondrial features, and applied multivariate approaches to develop an integrative model in which 7-KC acts as a signaling domain contributing to a leukemia-supportive, therapy-tolerant stromal state in AML.

## 2. Results

### 2.1. Cytotoxicity of 7-KC in HD-MSCs and AML-MSCs

**Figure 1:**
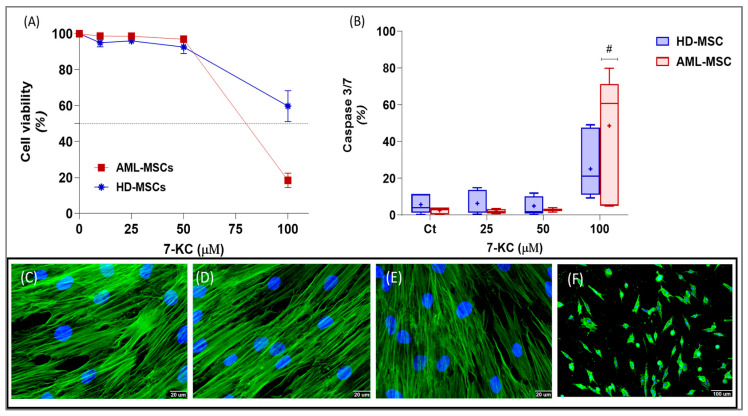
Effect of 7-KC on viability, apoptosis, and cytoskeleton in HD-MSCs and AML-MSCs (*n* = 5 cell lines/group; 24 h). (**A**) Cytotoxicity assessed by Hoechst 33342 and propidium iodide; nonlinear regression used for curve fitting and IC_50_ estimation. (**B**) Caspase-3/7 activation (%) (two-way ANOVA + Tukey: treatment *p* = 0.0002; lineage *p* = 0.1694; interaction *p* = 0.0972). Data shown as box-and-whisker plots (mean (+), median, IQR, min–max). # differences among treatments within the same lineage. Ct = control. (**C**–**F**) Representative F-actin staining (FITC, green) in HD-MSCs: Ct (**C**), 25 µM (**D**), 50 µM (**E**), 100 µM (**F**). Nuclei: Hoechst 33342 (DAPI, blue). Images acquired on a High-Content Screening platform using 40× ((**A**,**B**); scale bar, 20 µm) and 10× ((**C**–**F**); scale bar, 100 µm) objectives.

To initially assess the cellular response to 7-KC, we evaluated its cytotoxic effects in HD-MSCs and AML-MSCs at concentrations of 10 µM, 25 µM, 50 µM, and 100 µM. A clear difference in the mean inhibitory concentration (IC_50_) was observed between the two cell types: HD-MSCs exhibited an IC_50_ of 98.0 µM, whereas AML-MSCs presented a lower IC_50_ of 82.0 µM (Figure 1A), indicating the increased susceptibility of AML-MSCs to 7-KC.

Given that apoptotic signaling represents a major mechanism of cytotoxicity, we next examined whether 7-KC induced activation of caspase-3/7 in MSCs. Exposure to higher, cytotoxic concentrations of 7-KC significantly increased caspase-3/7 activity (*p* = 0.002). However, no significant differences were detected between HD-MSCs and AML-MSCs (*p* = 0.1694), and no interaction effect was observed between treatment and cell lineage (*p* = 0.0972). Notably, AML-MSCs treated with 100 µM 7-KC demonstrated a marked increase in caspase-3/7 activation compared with the control (*p* = 0.0003) and with the 25 µM (*p* = 0.0003) and 50 µM (*p* = 0.0004) treatment groups (Figure 1B), suggesting a lineage-specific threshold of susceptibility.

To further explore the cellular effects associated with 7-KC exposure, we investigated its impact on cytoskeletal organization by evaluating F-actin distribution through indirect immunofluorescence. In control MSCs, F-actin displayed a well-organized network distributed throughout the cytoplasm. In contrast, cells treated with 50 µM 7-KC exhibited a noticeable reduction in F-actin fiber formation, accompanied by decreased cell volume and a loss of defined cell borders. These alterations were even more pronounced at 100 µM, where F-actin integrity was markedly compromised and a substantial reduction in total cytoplasmic area was observed (Figure 1C–F). Together, these findings indicate that 7-KC disrupts cytoskeletal organization in a concentration-dependent manner.

### 2.2. Mitochondrial Function After 7-KC Treatment

The effect of 7-KC on the mitochondrial membrane potential of MSCs was evaluated using the fluorescence intensity of the TMRE dye. TMRE is a cationic, cell-permeable fluorescent probe that accumulates within active mitochondria; thus, red fluorescence indicates TMRE sequestration in polarized mitochondria in both MSC lineages.

**Figure 2:**
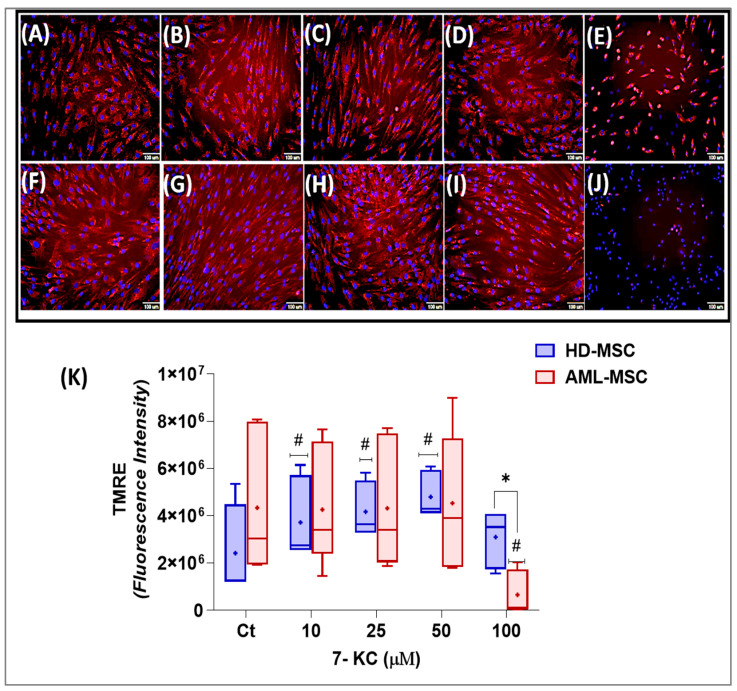
Effect of 7-KC on mitochondrial membrane potential in HD-MSCs and AML-MSCs (*n* = 5 cell lines/group; 24 h). (**A**–**J**) Representative TMRE images (TRITC, red): HD-MSCs (**A**–**E**) and AML-MSCs (**F**–**J**): basal (**A**,**F**) or 7-KC 10, 25, 50, 100 µM (**B**–**E**,**G**–**J**). Nuclei: Hoechst 33342 (DAPI, blue). Images acquired on a High-Content Screening platform using a 10× objective (scale bar, 100 µm). (**K**) TMRE fluorescence intensity quantification (two-way ANOVA + Tukey: treatment *p* = 0.0103; lineage *p* = 0.9724; interaction *p* < 0.0001). Data shown as box-and-whisker plots (mean (+), median, IQR, min–max). # differences among treatments within the same lineage; * differences between HD-MSCs and AML-MSCs under the same treatment.

Visual inspection revealed differences in staining patterns and mitochondrial architecture between HD-MSCs and AML-MSCs treated with 10, 25, 50 or 100 µM 7-KC (Figure 2). In basal medium, both MSC lineages displayed a homogeneous distribution of elongated, tubular mitochondria dispersed throughout the cytoplasm (Figure 2A,F).

Treatment with 25 µM 7-KC altered mitochondrial morphology, resulting in a reticular, network-like structure across the cytoplasm (Figure 2B). At 50 µM 7-KC, the previously homogeneous cytoplasmic distribution became less evident, with mitochondria showing a clear perinuclear translocation and a reduction in the reticular network (Figure 2C). At 100 µM 7-KC, the reticular mitochondrial structure was completely disrupted. In addition, some mitochondria lost the ability to retain TMRE, as indicated by visible nuclear staining (blue) in the absence of TMRE fluorescence. Under these conditions, mitochondria appeared rounded or punctate and were predominantly located in the perinuclear region (Figure 2E).

Based on TMRE fluorescence intensity, a statistical analysis was performed to determine the effect of 7-KC on the mitochondrial membrane potential of MSCs. No significant difference was observed between HD-MSCs and AML-MSCs cultured under basal conditions (*p* = 0.0830; Figure 2K). Two-way ANOVA revealed a significant effect of 7-KC concentration on TMRE fluorescence (*p* = 0.0103), as well as a significant interaction between treatment and MSC lineage (*p* < 0.0001). In AML-MSCs, TMRE intensity was significantly reduced at 100 µM 7-KC compared with the basal control (*p* < 0.0001), indicating mitochondrial membrane depolarization. In contrast, HD-MSCs showed increased TMRE fluorescence at 10–50 µM 7-KC relative to the control (*p* < 0.0001), a pattern consistent with mitochondrial membrane hyperpolarization. When comparing lineages, a significant difference in TMRE intensity was observed at 100 µM 7-KC (*p* < 0.0001): While HD-MSCs maintained mitochondrial polarization, AML-MSCs exhibited pronounced depolarization (Figure 2K).

### 2.3. Selection of Subtoxic 7-KC Concentrations Based on MTT and Cell Cycle Analyses

**Figure 3:**
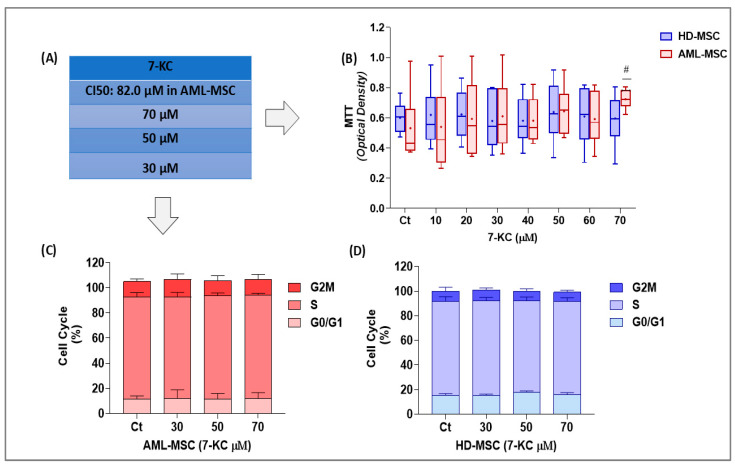
Effects of sub-IC_50_ 7-KC on metabolic activity and cell cycle in HD-MSCs and AML-MSCs (*n* = 5 cell line/group; 24 h). (**A**) Sub-IC_50_ 7-KC concentrations selected for subsequent experiments. (**B**) MTT reduction (optical density) (two-way ANOVA + Tukey: treatment *p* = 0.0363; lineage *p* = 0.9701; interaction *p* = 0.0136). # differences among treatments within the same lineage. (**C**,**D**) Cell-cycle phase percentage; two-way ANOVA + Tukey showed no effects of treatment, lineage, or interaction (all *p* > 0.0500).

Based on the cytotoxicity results, concentrations below the IC50 were selected for subsequent experiments (Figure 3A). Because the IC50 of 7-KC was lower in AML-MSCs (82.0 µM) than in HD-MSCs, the sub-IC50 concentrations were defined using AML-MSC sensitivity as the reference, ensuring cell viability for downstream analyses. We selected the following concentrations: 30 µM, 50 µM, and 70 µM of 7-KC.

To determine whether these concentrations could induce functional alterations in MSCs, two complementary assays were performed: the MTT assay and cell cycle analysis, in both HD-MSCs and AML-MSCs.

The MTT assay was performed using seven concentrations below the IC50 of 7-KC in both MSC lineages treated for 24 h, assessing the conversion of MTT into formazan by cellular oxidoreductases. Treatment with 7-KC significantly affected MTT reduction (*p* = 0.0363). Although no differences were observed between HD-MSCs and AML-MSCs (*p* = 0.9701), a significant interaction between treatment and cell lineage was detected (*p* = 0.0136). This effect occurred in AML-MSCs, specifically between the control and the 70 µM treatment (*p* = 0.0013) (Figure 3B).

Cell cycle analysis was performed to assess whether 7-KC treatment for 24 h exerted a cytostatic effect on MSCs. No significant alterations in the cell cycle profile were observed in either HD-MSCs or AML-MSCs at the tested concentrations (Figure 3C,D).

### 2.4. Comprehensive Evaluation of ABC Transporters: Global Gene Profiling Followed by Protein-Level Validation of Multidrug Resistance Markers

**Figure 4:**
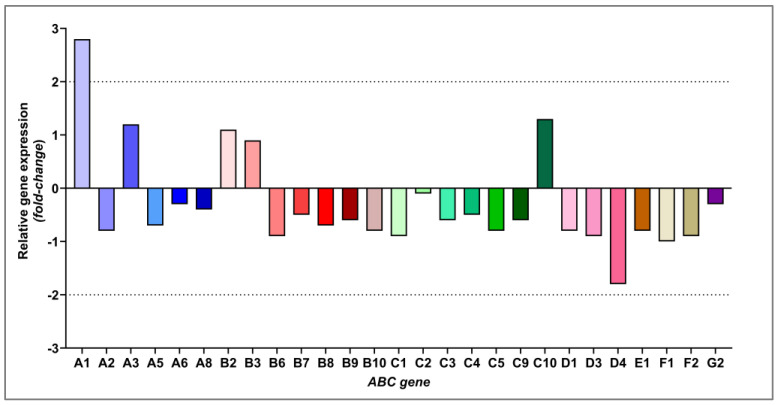
Relative gene expression of ABC transporters in an AML-MSC line treated with 25 µM 7-KC after 24 h, shown as fold-change relative to basal medium. The dashed line indicates a 2-fold-change threshold.

To select which ABC transporters would be investigated at the protein level, we first performed a broad assessment of the relative expression of 44 genes belonging to the ABC superfamily. The AML-MSC lineage was treated with 25 µM 7-KC for 24 h, and gene expression values were compared with those of the control cultured in basal medium. The overall distribution of fold-changes allowed the identification of general patterns of positive and negative modulation among the different ABC subfamilies (Figure 4).

Within the ABCA subfamily, the genes ABCA1, ABCA2, ABCA3, ABCA5, and ABCA8 showed changes in relative expression after 7-KC treatment. Among the ABCB genes, the modulation of ABCB2, ABCB3, ABCB6, ABCB7, ABCB8, ABCB9, and ABCB10 was observed. In the ABCC subfamily, alterations were detected in ABCC1, ABCC2, ABCC3, ABCC4, ABCC5, ABCC9, and ABCC10. For the ABCD genes, ABCD1, ABCD3, and ABCD4 showed changes in expression. Finally, in the ABCE/F subfamily, ABCE1, ABCF1, and ABCF2 were modulated, whereas in the ABCG family, ABCG2 varied after 7-KC treatment.

Although some of the changes in gene expression were of low magnitude, these results were integrated with widely recognized biological and functional criteria. Thus, the selection of genes for protein-level validation was based both on the magnitude of relative expression and on the physiological relevance of each ABC transporter. In this way, priority was given to genes related to multidrug resistance, lipid homeostasis, or the transport of cholesterol-derived molecules.

In this context, in addition to differentially modulated genes, the ABCG1 gene, whose expression did not show a significant change after 7-KC treatment, was included. The choice of ABCG1 is due to its key role in reverse cholesterol transport, intracellular lipid homeostasis, and the efflux of oxysterols, including molecules structurally related to 7-KC. Thus, six ABC proteins were selected for protein-level validation by immunofluorescence: ABCA1, ABCC1, ABCC2, ABCD4, ABCG1, and ABCG2.

#### 2.4.1. ABCA1, ABCC1, ABCC2, ABCG1, ABCG2 and LRPs

**Figure 5:**
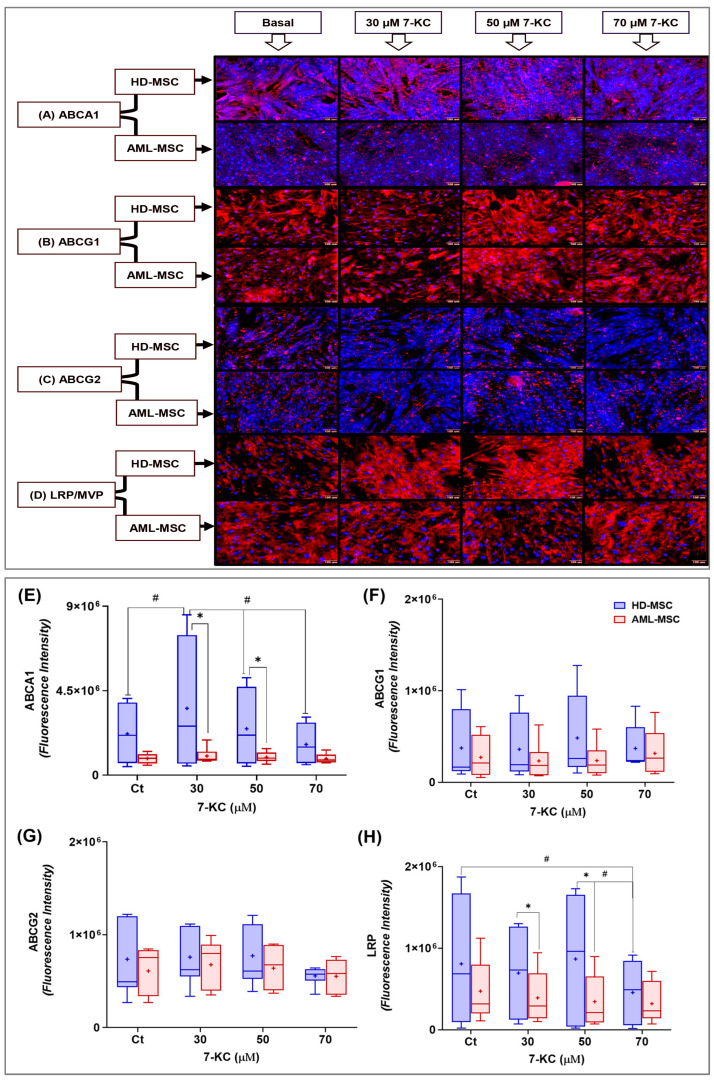
Effect of 7-KC on ABCA1, ABCG1, ABCG2 and LRPs in HD-MSCs and AML-MSCs (*n* = 5 cell lines/group; 24 h). (**A**–**D**) Representative immunolabeling (TRITC, red) of (**A**) ABCA1, (**B**) ABCG1, (**C**) ABCG2, and (**D**) LRP in HD-MSCs and AML-MSCs treated with 0, 30, 50, or 70 µM 7-KC. Nuclei: Hoechst 33342 (DAPI/blue). Cell boundaries: CellMask™ Blue (ABCA1 and ABCG2). Images acquired on a High-Content Screening platform using a 10× objective (scale bar, 100 µm). (**E**–**H**) Fluorescence intensity quantification of (**E**) ABCA1 (two-way ANOVA + Tukey: treatment *p* = 0.0008; lineage *p* = 0.0018; interaction *p* = 0.0148), (**F**) ABCG1 (treatment *p* = 0.4240; lineage *p* = 0.3542; interaction *p* = 0.0251), (**G**) ABCG2 (treatment *p* = 0.0116; lineage *p* = 0.9616; interaction *p* = 0.9151), and (**H**) LRP (treatment *p* = 0.0023; lineage *p* = 0.0663; interaction *p* = 0.0284). Data shown as box-and-whisker plots (mean (+), median, IQR, min–max). # differences among treatments within the same lineage; * differences between HD-MSCs and AML-MSCs under the same treatment.

ABCA1 is a transmembrane protein that can be observed as punctate red staining at the cell plasma membrane borders, which are delimited by the light-blue labeling (Figure 5A). Visual inspection of the images revealed differences in fluorescence intensity between HD-MSCs and AML-MSCs. In addition, HD-MSCs treated with 30 µM 7-KC and 50 µM 7-KC showed higher fluorescence intensity, with an evident increase in ABCA1 labeling at the plasma membrane borders.

Based on ABCA1 fluorescence intensity, we compared HD-MSCs and AML-MSCs. Analysis of the effect of 7-KC treatment showed a significant difference in ABCA1 levels among the MSCs (treatment effect: *p* = 0.0008), between lineages (lineage effect: *p* = 0.0018), and a significant interaction between 7-KC treatment and cell lineage response (interaction: *p* = 0.0148; Figure 5E). This differential response in ABCA1 levels was observed in HD-MSCs, in which treatment with 30 µM 7-KC increased ABCA1 compared with cells cultured in basal medium (*p* = 0.0098). ABCA1 fluorescence intensity at 50 µM and 70 µM 7-KC was similar to basal control (*p* > 0.0500), but significantly lower than at 30 µM 7-KC (*p* = 0.0399 and *p* = 0.0004, respectively). When comparing the response between HD-MSCs and AML-MSCs, 30 µM and 50 µM 7-KC increased ABCA1 in HD-MSCs, whereas no significant changes were observed in AML-MSCs (*p* < 0.0500). Moreover, at all 7-KC concentrations tested, no effect on ABCA1 levels was detected in AML-MSCs (Figure 5E).

ABCG1 is a transmembrane protein that was observed as a diffuse red staining throughout the cell body, with a predominantly cytoplasmic and membranous distribution and no apparent nuclear localization (Figure 5B). The fluorescence signal appeared relatively homogeneous among cells, without evident clustering or punctate structures. Here, no major qualitative differences were evident between treatments by visual inspection. Treatment with 7-KC showed a significant interaction between MSC lineage and 7-KC concentration (*p* = 0.0251), with a difference observed at 50 µM 7-KC, where ABCG1 levels were increased in HD-MSCs compared with AML-MSCs (*p* = 0.0002; Figure 5F). Although this difference reached statistical significance, it is important to note that this result was strongly influenced by the dispersion of the observed values.

ABCG2 was detected as punctate red staining distributed throughout the cell body, often concentrated near the cell periphery (Figure 5C). The intensity of the red fluorescence is proportional to the amount of ABCG2 protein in HD-MSCs and AML-MSCs. In the overall variance analysis, 7-KC treatment significantly affected ABCG2 levels in both HD-MSCs and AML-MSCs, with a trend toward reduced ABCG2 fluorescence at 70 µM 7-KC (treatment effect: *p* = 0.0116). However, Tukey’s post hoc pairwise comparisons did not identify any statistically significant differences between individual treatment groups (*p* > 0.0500; Figure 5G). Two-way ANOVA showed no significant effect of cell lineage (*p* = 0.9616) and no treatment × lineage interaction (*p* = 0.9151), as seen in Figure 5G.

By visual inspection, LRP showed a diffuse, granular cytoplasmic staining pattern, predominantly perinuclear, with no evident nuclear labeling in either lineage (Figure 5D). Qualitatively, fluorescence intensity appeared higher in HD-MSCs than in AML-MSCs. Across the different 7-KC concentrations, no major qualitative changes were observed, although an apparent reduction in LRP signal was noted in HD-MSCs exposed to 70 µM 7-KC. Treatment with 7-KC had a significant effect on LRP levels (*p* = 0.0023), with an interaction between treatment and MSC lineage (*p* = 0.0284). In AML-MSCs, LRP levels remained stable across groups (*p* > 0.0500). By contrast, in HD-MSCs, 70 µM 7-KC reduced LRP levels compared with 50 µM 7-KC (*p* = 0.0013) and with basal medium control (*p* = 0.0068). When comparing lineages, differences between HD-MSCs and AML-MSCs were observed at 30 µM (*p* = 0.0248) and 50 µM (*p* < 0.0001) 7-KC (Figure 5H).

ABCC1 and ABCC2 proteins were evaluated by indirect immunofluorescence in both HD-MSC and AML-MSC lineages treated with 7-KC for 24 h. However, no expression of these proteins was detected in either HD-MSCs or AML-MSCs under these experimental conditions.

#### 2.4.2. ABCD4 Protein

**Figure 6:**
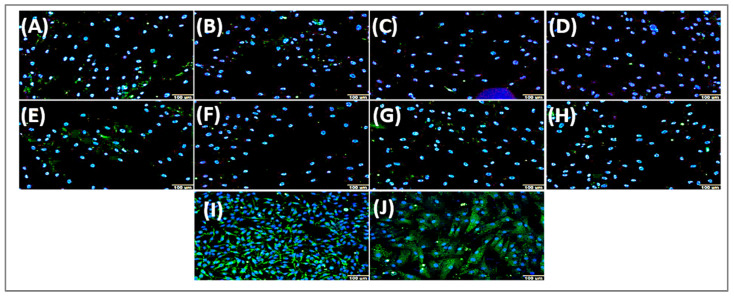

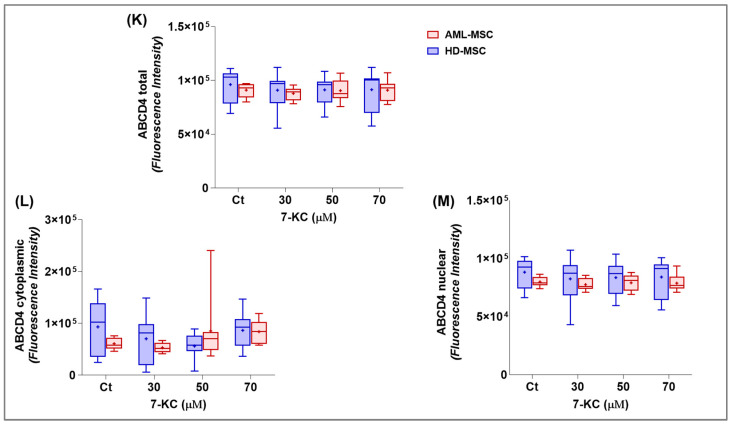
ABCD4 expression in MSCs treated with 7-KC and in reference cell lines (*n* = 5 MSC lines/group; 24 h). (**A**–**H**) Representative ABCD4 immunolabeling (FITC, green): HD-MSCs (**A**–**D**) and AML-MSCs (**E**–**H**) treated with 0, 30, 50, or 70 µM 7-KC; (**I**,**J**) ABCD4 cytoplasmic staining in MDA-MB-231 cells (**I**) and MRC-5 fibroblasts (**J**). Nuclei: Hoechst 33342 (DAPI, blue). Images acquired on a High-Content Screening platform using a 10× objective (scale bar, 100 µm). (**K**–**M**) Total, cytoplasmic, and nuclear ABCD4 levels in MSCs. Two-way ANOVA + Tukey: total—treatment *p* = 0.3188, lineage *p* = 0.7745, interaction *p* = 0.3439; cytoplasmic—treatment *p* = 0.3662, lineage *p* = 0.6954, interaction *p* = 0.1160; nuclear—treatment *p* = 0.1442, lineage *p* = 0.4529, interaction *p* = 0.6208. Data shown as box-and-whisker plots (mean (+), median, IQR, min–max).

Visual inspection of the photomicrographs revealed the presence of ABCD4 in the nuclei of mesenchymal stem cells, showing a punctate, speckled green nuclear staining pattern in both MSC lineages. In the cytoplasm, ABCD4 labeling was also observed as a discrete green point (Figure 6). This combined nuclear and cytoplasmic pattern of ABCD4 in bone marrow MSCs was unexpected, as ABCD4 has predominantly been reported in non-nuclear compartments, suggesting a lineage-specific subcellular distribution in these cells. To confirm the specific binding of the anti-ABCD4 antibody and the subcellular localization of ABCD4, indirect immunofluorescence was performed in MDA-MB-231 (breast adenocarcinoma) and MRC-5 (human fetal lung fibroblast) cell lines, in which ABCD4 staining was restricted to the cytoplasm and no nuclear labeling was detected (Figure 6I,J). Based on the previous imaging results, we next quantified total, cytoplasmic, and nuclear ABCD4 in HD-MSCs and AML-MSCs treated with 7-KC. Treatment with the oxysterol 7-KC for 24 h did not significantly alter total ABCD4 levels between MSC lineages (*p* = 0.3188; Figure 6K). Regarding cytoplasmic ABCD4, a trend toward differences in concentration was observed, but it did not reach statistical significance (*p* = 0.3662; Figure 6L), and nuclear ABCD4 levels were likewise not significantly different between groups (*p* = 0.1442; Figure 6M).

### 2.5. 7-Ketocholesterol-Induced Modulation of Signaling Pathways in HD-MSCs and AML-MSCs

Considering that 7-KC acts both as a cytotoxic oxysterol and as a modulator of signaling pathways involved in lipid homeostasis, stress response, and stem cell behavior, in the next step we evaluated molecular targets related to these processes in HD-MSCs and AML-MSCs. We investigated classical pathways controlling cholesterol/oxysterol metabolism (LXR), maintenance of the stem cell and niche phenotype (Hedgehog/SMO/GLI3), and axes associated with stress response and cell survival (CD147, survivin, NF-κB), in addition to parameters of redox status and mitochondrial function.

#### 2.5.1. LXR-α/β, PPAR-γ and Caveolin-1 Signaling

We evaluated whether 7-KC treatment alters the levels of the nuclear receptors LXR-α and LXR-β in MSC lineages by indirect immunofluorescence.

**Figure 7:**
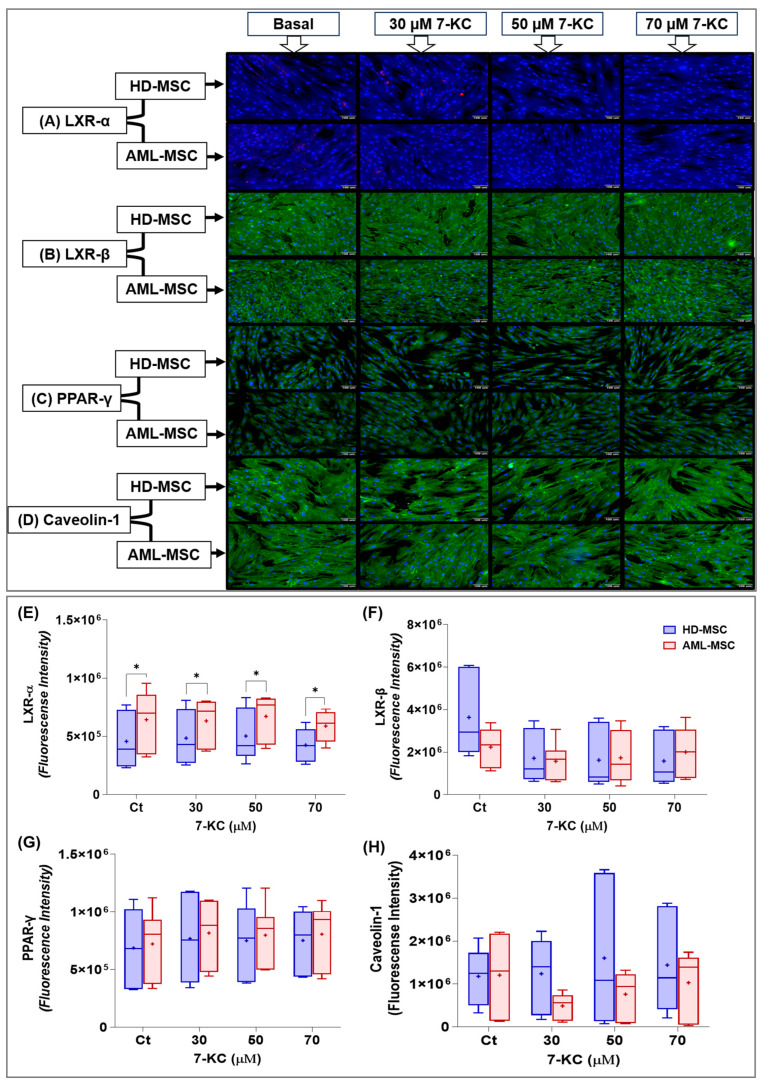
LXR-α, LXR-β, PPAR-γ and caveolin-1 in MSCs treated with 7-KC (*n* = 5 cell lines/group; 24 h). Representative immunofluorescence images of (**A**) LXR-α (TRITC—red and CellMask™ Blue), (**B**) LXR-β (FITC—green), (**C**) PPAR-γ (FITC—green) and (**D**) caveolin-1 (FITC—green) in HD-MSCs and AML-MSCs cultured in basal medium or treated with 30, 50 or 70 µM 7-KC. Nuclei were stained with Hoechst 33342 (DAPI—blue); images were acquired using a High-Content Screening platform with a 10× objective; scale bar = 100 µm. Quantitative analysis of fluorescence intensity with two-way ANOVA + Tukey: (**E**) LXR-α—treatment *p* = 0.2207, lineage *p* = 0.0192, interaction *p* = 0.7605; (**F**) LXR-β—treatment *p* = 0.0750, lineage *p* = 0.3375, interaction *p* = 0.1935; (**G**) PPAR-γ—treatment *p* = 0.0509, lineage *p* = 0.7718, interaction *p* = 0.9910; (**H**) caveolin-1—treatment *p* = 0.7886, lineage *p* = 0.0958, interaction *p* = 0.7112. Data shown as box-and-whisker plots (mean (+), median, IQR, min–max). * differences between HD-MSCs and AML-MSCs under the same treatment.

LXR-α showed a punctate red staining pattern mainly in the cytoplasm, with a predominantly perinuclear distribution in MSCs (Figure 7A). By visual inspection, fluorescence intensity appeared higher in AML-MSCs cultured in basal medium compared with HD-MSCs. LXR-β showed a diffuse green staining pattern throughout the cytoplasm of MSCs, with a predominantly perinuclear distribution and no clear evidence of nuclear labeling (Figure 7B).

Visual inspection of the photomicrographs revealed PPAR-γ as a punctate green staining pattern distributed mainly throughout the cytoplasm, with a predominantly perinuclear localization and only faint nuclear labeling in some cells (Figure 7C). In addition to the nuclear receptors LXR and PPAR-γ, we evaluated caveolin-1 as a structural protein associated with cholesterol-enriched membrane microdomains, potentially involved in the organization of signaling complexes activated by 7-KC.

Caveolin-1 is associated with the plasma membrane and cholesterol-rich membrane microdomains and can also localize to the Golgi complex and caveolar vesicles. Visual inspection of the photomicrographs revealed caveolin-1 as a green, diffuse staining pattern throughout the cytoplasm, following the cell contour and consistent with plasma membrane labeling (Figure 7D).

The 7-KC treatment did not have a significant effect on LXR-α levels (treatment effect: *p* = 0.2207), and no significant treatment × lineage interaction was detected (*p* = 0.7605). Differences were observed only between HD-MSCs and AML-MSCs, regardless of 7-KC concentration (cell lineage effect: *p* = 0.0192; Figure 7E).

Although 7-KC treatment for 24 h tended to reduce LXR-β levels in both MSC lineages, this effect did not reach statistical significance and remained borderline (treatment effect: *p* = 0.0750; Figure 7F). No significant differences were detected between HD-MSCs and AML-MSCs (cell lineage effect: *p* = 0.3375), and there was no significant treatment × lineage interaction (*p* = 0.1935).

Overall, 7-KC treatment did not significantly affect PPAR-γ levels (treatment effect: *p* = 0.0509), although a borderline trend was observed (Figure 7G). No significant differences were found between HD-MSCs and AML-MSCs (cell lineage effect: *p* = 0.7718), and there was no treatment × lineage interaction (*p* = 0.9910), as seen in Figure 7G.

Statistical analysis showed that caveolin-1 levels were similar between HD-MSCs and AML-MSCs (cell lineage effect: *p* = 0.0958), with only a slight, non-significant trend toward higher values in HD-MSCs (Figure 7H). Although mean caveolin-1 levels appeared to vary across 7-KC treatment groups, two-way ANOVA revealed no significant effect of treatment (*p* = 0.7886) and no treatment × lineage interaction (*p* = 0.7112), likely due to the high dispersion of fluorescence intensity values (Figure 7H).

To further explore alternative signaling mechanisms potentially involved in the response of MSCs to 7-KC, we next investigated components of the Hedgehog/SMO/GLI3 pathway.

#### 2.5.2. Hedgehog, SMO, and GLI3 Signaling

**Figure 8:**
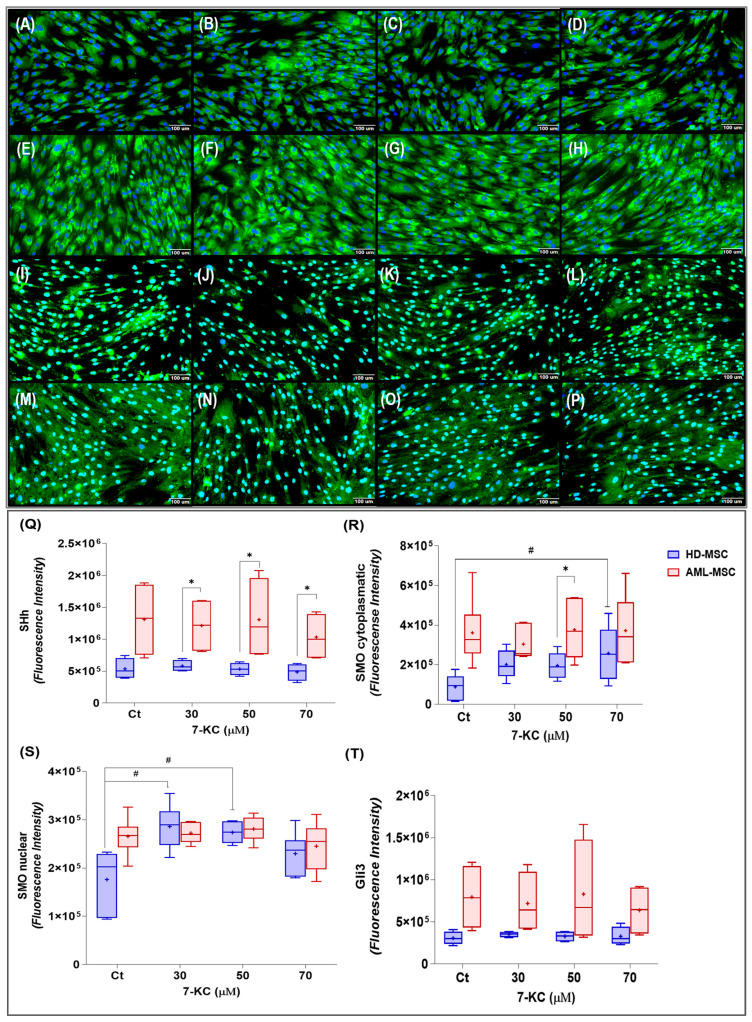
SHH pathway components in HD-MSCs and AML-MSCs treated with 7-KC (*n* = 5 cell lines/group). (**A**–**H**) Representative SHH immunolabeling (FITC, green) in HD-MSCs (**A**–**D**) and AML-MSCs (**E**–**H**) treated with 0, 30, 50, or 70 µM 7-KC. (**I**–**P**) Representative SMO immunolabeling (FITC, green) in HD-MSCs (**I**–**L**) and AML-MSCs (**M**–**P**) treated with 0, 30, 50, or 70 µM 7-KC. Nuclei: Hoechst 33342 (DAPI, blue). Images acquired on a High-Content Screening platform using a 10× objective (scale bar, 100 µm). (**Q**–**T**) Fluorescence intensity quantification of (**Q**) SHH, (**R**) cytoplasmic SMO, (**S**) nuclear SMO, and (**T**) GLI3 (two-way ANOVA + Tukey: SHH—treatment *p* = 0.0699, lineage *p* = 0.1152, interaction *p* = 0.2040; cytoplasmic SMO—treatment *p* = 0.2521, lineage *p* = 0.0458, interaction *p* = 0.1224; nuclear SMO—treatment *p* = 0.0079, lineage *p* = 0.0311, interaction *p* = 0.0642; GLI3—treatment *p* = 0.6988, lineage *p* = 0.1524, interaction *p* = 0.5931). Data shown as box-and-whisker plots (mean (+), median, IQR, min–max). # differences among treatments within the same lineage; * differences between HD-MSCs and AML-MSCs under the same treatment.

In this study, Sonic Hedgehog (SHH) was evaluated by indirect immunofluorescence in HD-MSCs and AML-MSCs treated with 7-KC for 24 h. In the photomicrographs, SHH appears as a green, diffuse and punctate staining throughout the cytoplasm, with areas of perinuclear accumulation in both lineages. By visual inspection, SHH fluorescence intensity seems higher in AML-MSCs than in HD-MSCs (Figure 8A–H).

SMO is a transmembrane receptor of the Hedgehog pathway, primarily localized at the plasma membrane and intracellular compartments. Visual inspection of the photomicrographs revealed SMO as a green punctate and diffuse staining pattern in both HD-MSCs and AML-MSCs, distributed throughout the cytoplasm with perinuclear accumulation, without a clearly defined continuous plasma membrane ring. In some cells, partial overlap of the signal with the nuclear region was observed (Figure 8J–P).

For SHH, 24 h treatment with 7-KC had no significant effect in either HD-MSCs or AML-MSCs (treatment factor: *p* = 0.0699; cell lineage factor: *p* = 0.1152; interaction: *p* = 0.2040), although a trend toward higher SHH levels was observed in AML-MSCs compared with HD-MSCs (Figure 8Q).

For cytoplasmic SMO, two-way ANOVA did not show a significant effect of 7-KC treatment (*p* = 0.2521), but indicated an effect of cell lineage (*p* = 0.0458), with higher values in AML-MSCs than in HD-MSCs and no significant interaction (*p* = 0.1224). When lineages were analyzed separately, one-way ANOVA in HD-MSCs revealed an effect of 7-KC treatment on cytoplasmic SMO (*p* = 0.0364), with a significant increase at 70 µM compared with basal control (*p* = 0.0244). In AML-MSCs, no significant differences were observed between treatment groups (*p* > 0.0500) (Figure 8R).

For nuclear SMO, 24 h treatment with 7-KC significantly increased protein levels (treatment factor: *p* = 0.0079), and the response to 7-KC differed between lineages (cell lineage factor: *p* = 0.0311; treatment × lineage interaction borderline: *p* = 0.0642). This effect was mainly observed in HD-MSCs, in which nuclear SMO increased at 30 µM (*p* = 0.0128) and 50 µM (*p* = 0.0314) 7-KC compared with basal control. In AML-MSCs, no significant differences were detected between treatment groups (*p* > 0.0500) (Figure 8S).

For GLI3, a repressor of the SHH pathway, 7-KC treatment did not significantly change its levels in either MSC lineage (treatment factor: *p* = 0.6988), with no effect of cell lineage (*p* = 0.1524) or interaction (*p* = 0.5931) (Figure 8T).

These findings indicate that 7-KC does not markedly change SHH or GLI3 levels, but selectively enhances SMO, particularly its nuclear pool, in HD-MSCs, while AML-MSCs remain largely unresponsive. This pattern is consistent with a partial and lineage-dependent rewiring of Hedgehog signaling rather than a full pathway activation.

#### 2.5.3. Stress- and Survival-Related Signaling

Given that 7-KC is also known to trigger stress and pro-survival responses, we next examined the membrane marker CD147 and survivin, which was quantified separately in the cytoplasmic and nuclear compartments. In addition, we evaluated the gene expression of NF-κB and BCL2 as key components of a stress- and survival-associated signaling network in HD-MSCs and AML-MSCs.

**Figure 9:**
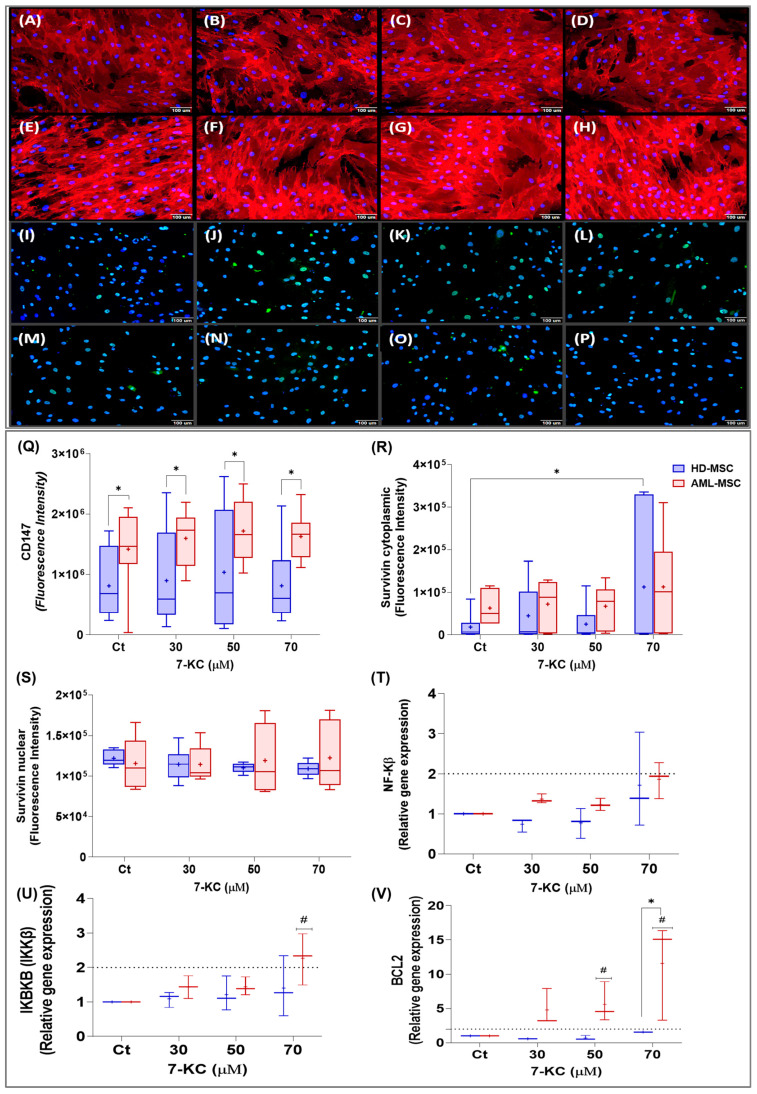
CD147, survivin, and NF-κB pathway markers in HD-MSCs and AML-MSCs treated with 7-KC (*n* = 5 cell lines/group; 24 h). (**A**–**H**) Representative CD147 immunolabeling (TRITC, red) in HD-MSCs (**A**–**D**) and AML-MSCs (**E**–**H**) treated with 0, 30, 50, or 70 µM 7-KC. (**I**–**P**) Representative survivin immunolabeling (FITC, green) in HD-MSCs (**I**–**L**) and AML-MSCs (**M**–**P**) treated with 0, 30, 50, or 70 µM 7-KC. Nuclei: Hoechst 33342 (DAPI, blue). Images acquired on a High-Content Screening platform using a 10× objective (scale bar, 100 µm). (**Q**–**V**) Quantification of (**Q**) CD147, (**R**) cytoplasmic survivin, (**S**) nuclear survivin and relative mRNA expression of (**T**) NF-κB, (**U**) IKBKB (IKKβ), and (**V**) BCL2 (two-way ANOVA + Tukey: CD147—treatment *p* = 0.1695, lineage *p* = 0.0905, interaction *p* = 0.8391; cytoplasmic survivin—treatment *p* = 0.1753, lineage *p* = 0.2921, interaction *p* = 0.4704; nuclear survivin—treatment *p* = 0.8430, lineage *p* = 0.8012, interaction *p* = 0.2372; NF-κB—treatment *p* = 0.0405, lineage *p* = 0.0007, interaction *p* = 0.1070; IKBKB—treatment *p* = 0.0491, lineage *p* = 0.0834, interaction *p* = 0.4672; BCL2—treatment *p* = 0.0297, lineage *p* = 0.0010, interaction *p* = 0.0628). Data shown as box-and-whisker plots (mean (+), median, IQR, min–max). # differences among treatments within the same lineage; * differences between HD-MSCs and AML-MSCs under the same treatment.

CD147 is a transmembrane protein associated with the plasma membrane. Visual inspection of the photomicrographs shows CD147 labeling in red, with a continuous, reticular pattern along the cell contour, consistent with plasma membrane staining in MSCs (Figure 9A–H).

CD147 levels were analyzed in HD-MSCs and AML-MSCs treated with different concentrations of 7-KC for 24 h. Two-way ANOVA showed no significant effect of 7-KC treatment on CD147 (treatment factor: *p* = 0.1695), and no significant cell lineage effect (*p* = 0.0905) or interaction (*p* = 0.8391), although AML-MSCs tended to display higher fluorescence values than HD-MSCs across conditions (Figure 9Q).

Survivin is a member of the inhibitor of apoptosis (IAP) family that regulates cell survival and mitosis, and can be found in both the cytoplasm and nucleus. In the images, survivin appears as green staining with a diffuse punctate pattern in the cytoplasm and a punctate pattern in the nucleus in both MSC lineages (Figure 9I–P).

Based on fluorescence intensity, 7-KC treatment for 24 h increased cytoplasmic survivin in HD-MSCs at 70 µM compared with basal control (*p* = 0.0099; Figure 9R). However, in the two-way ANOVA including both lineages, no significant main effect of treatment (*p* = 0.1753), cell lineage (*p* = 0.2921), or interaction (*p* = 0.4704) was detected. In contrast, nuclear survivin levels were not significantly affected by 7-KC in either lineage (treatment factor: *p* = 0.8430; cell lineage factor: *p* = 0.8012; interaction: *p* = 0.2372), as seen in Figure 9S.

At the transcriptional level, 7-KC increased NF-κB gene expression (treatment factor: *p* = 0.0405), and AML-MSCs exhibited higher NF-κB mRNA levels than HD-MSCs (cell lineage factor: *p* = 0.0007), with no significant treatment with lineage interaction (*p* = 0.1070), as seen in Figure 9T.

For IKBKB, two-way ANOVA revealed a significant treatment effect (*p* = 0.0491) and a trend toward higher expression in AML-MSCs (cell lineage factor: *p* = 0.0834), without a significant interaction (*p* = 0.4672). Post hoc analysis showed that IKBKB expression was increased in AML-MSCs treated with 70 µM 7-KC compared with basal control (*p* = 0.0491), whereas no significant differences were observed among treatment groups in HD-MSCs (*p* > 0.0500), as seen in Figure 9V.

Finally, 7-KC also modulated the expression of the anti-apoptotic gene BCL2. Two-way ANOVA showed a significant effect of treatment (*p* = 0.0297) and higher BCL2 mRNA levels in AML-MSCs than in HD-MSCs (cell lineage factor: *p* = 0.0010), with a borderline treatment × lineage interaction (*p* = 0.0628). Post hoc analysis revealed that BCL2 expression was significantly increased in AML-MSCs treated with 70 µM 7-KC compared with basal control (*p* = 0.0011), whereas no significant treatment-related differences were observed among HD-MSC groups (*p* > 0.0500), as seen in Figure 9U.

Together, these data indicate that 7-KC reinforces a stress- and survival-oriented profile, particularly in AML-MSCs. We therefore next asked whether these responses were accompanied by alterations in cellular redox balance, focusing on glutathione metabolism.

#### 2.5.4. Glutathione Metabolism and Redox-Related Signaling

Because stress and survival pathways are tightly coupled to the cellular redox state, we subsequently evaluated glutathione metabolism in HD-MSCs and AML-MSCs exposed to 7-KC, focusing on reduced glutathione (GSH), oxidized glutathione (GSSG), and the GSH/GSSG ratio as indicators of redox balance.

**Figure 10:**
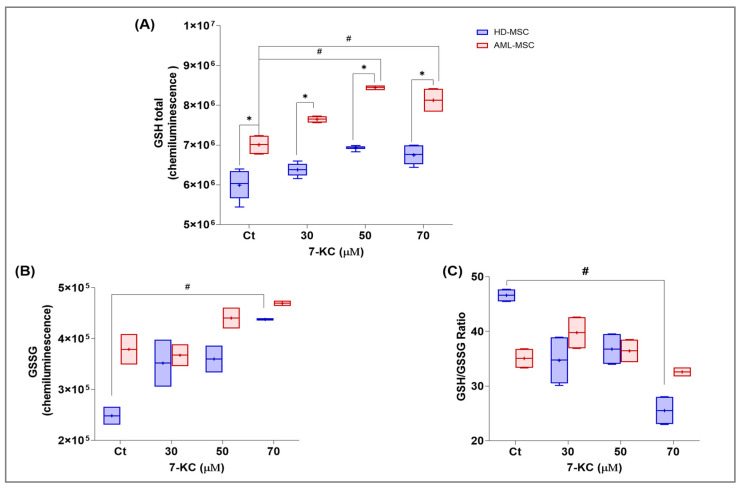
Glutathione metabolism in HD-MSCs and AML-MSCs treated with 7-KC (*n* = 5 cell lines/group; 24 h). (**A**) Total glutathione (GSH) and (**B**) oxidized glutathione (GSSG) levels (luminescence intensity), and (**C**) GSH/GSSG ratio after 24 h 7-KC (two-way ANOVA + Tukey: GSH—treatment *p* = 0.0013, lineage *p* < 0.0001, interaction *p* = 0.5899; GSSG—treatment *p* = 0.0028, lineage *p* = 0.0062, interaction *p* = 0.1660; GSH/GSSG—treatment *p* = 0.0080, lineage *p* = 0.9821, interaction *p* = 0.0209). Data shown as box-and-whisker plots (mean (+), median, IQR, min–max). # differences among treatments within the same lineage; * differences between HD-MSCs and AML-MSCs under the same treatment.

7-KC treatment for 24 h significantly increased total glutathione levels (treatment factor: *p* = 0.0013), and AML-MSCs displayed higher total GSH than HD-MSCs (cell lineage factor: *p* < 0.0001), with no significant treatment × lineage interaction (*p* = 0.5899). Post hoc analysis showed that, in AML-MSCs, total GSH was higher at 50 µM (*p* = 0.0096) and 70 µM (*p* = 0.0388) 7-KC compared with basal control. When lineages were compared within each treatment group, AML-MSCs exhibited higher total GSH than HD-MSCs at 30 µM (*p* = 0.0205), 50 µM (*p* = 0.0076) and 70 µM (*p* = 0.0135) 7-KC (Figure 10A).

Exposure to 7-KC for 24 h also increased oxidized glutathione (GSSG) levels (treatment factor: *p* = 0.0028), with higher GSSG in HD-MSCs than in AML-MSCs (cell lineage factor: *p* = 0.0062) and no significant interaction (*p* = 0.1660). The main change was observed in HD-MSCs treated with 70 µM 7-KC, which showed higher GSSG compared with basal control (*p* = 0.0087), as seen in Figure 10B.

We calculated the GSH/GSSG ratio as an index of redox balance. 7-KC treatment significantly decreased the GSH/GSSG ratio (treatment factor: *p* = 0.0080), and a significant treatment × lineage interaction was detected (*p* = 0.0209), whereas no main effect of cell lineage was observed (*p* = 0.9821). This decrease in the GSH/GSSG ratio was mainly driven by HD-MSCs treated with 70 µM 7-KC, which showed a lower ratio compared with basal control (*p* = 0.0041), while no significant differences between treatment groups were detected in AML-MSCs (*p* > 0.0500), as seen in Figure 10C.

Altogether, the increase in total and oxidized glutathione and the consequent reduction in the GSH/GSSG ratio indicate that 7-KC disrupts redox homeostasis in a lineage-dependent manner. We therefore next investigated whether these redox alterations were accompanied by changes in mitochondria-linked stress and dynamics-related gene expression.

#### 2.5.5. Mitochondria-Linked Stress and Dynamics-Related Gene Expression

Mitochondria are central integrators of redox homeostasis and cell fate decisions. To explore whether 7-KC-induced stress signaling was associated with changes in mitochondrial dynamics and activity, we analyzed the gene expression of key fusion-related proteins (MFN1, MFN2, OPA1) and the mitochondrial-encoded cytochrome b (CYTB and ND1) in HD-MSCs and AML-MSCs.

Mitochondrial dynamics-related genes were analyzed in HD-MSCs and AML-MSCs treated with 7-KC for 24 h, and relative expression was calculated using the ΔΔCt method (basal control = 1).

**Figure 11:**
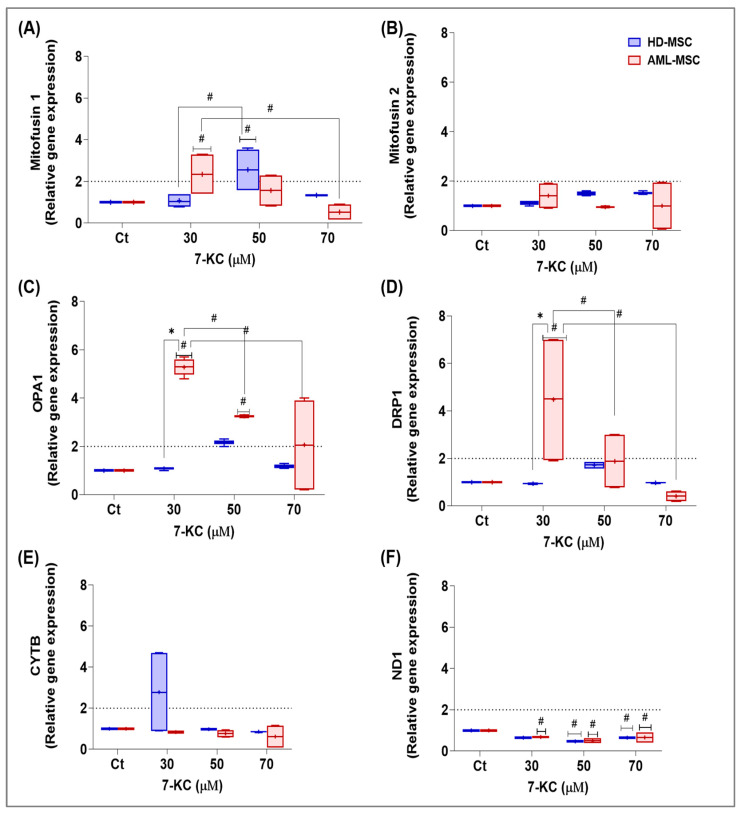
Mitochondria dynamics and electron transport chain gene expression in HD-MSCs and AML-MSCs treated with 7-KC (*n* = 5 cell lines/group; 24 h). Relative mRNA levels of (**A**) MFN1, (**B**) MFN2, (**C**) OPA1, (**D**) DRP1, (**E**) CYTB, and (**F**) ND1, shown as fold-change vs. basal control (set to 1) (two-way ANOVA ± Tukey: MFN1—treatment *p* = 0.0045, lineage *p* = 0.5646, interaction *p* = 0.0089; MFN2—treatment *p* = 0.5578, lineage *p* = 0.2118, interaction *p* = 0.1812; OPA1—treatment *p* < 0.0001, lineage *p* < 0.0001, interaction *p* < 0.0001; DRP1—treatment *p* = 0.0054, lineage *p* = 0.0528, interaction *p* = 0.0040; CYTB—treatment *p* = 0.0751, lineage *p* = 0.0670, interaction *p* = 0.1032; ND1—treatment *p* < 0.0001, lineage *p* = 0.7495, interaction *p* = 0.9957). Data shown as box-and-whisker plots (mean (+), median, IQR, min–max). # differences among treatments within the same lineage; * differences between HD-MSCs and AML-MSCs under the same treatment.

For *MFN1*, two-way ANOVA revealed a significant treatment effect (*p* = 0.0045), with no main effect of cell lineage (*p* = 0.5646) but a significant treatment × lineage interaction (*p* = 0.0089). In AML-MSCs, 70 µM 7-KC significantly decreased MFN1 expression compared with basal control (*p* = 0.0127), whereas 30 µM 7-KC showed only a trend toward increased expression (*p* = 0.0877). In HD-MSCs, 50 µM 7-KC significantly increased MFN1 expression relative to both basal control (*p* = 0.0125) and 30 µM 7-KC (*p* = 0.0183), as seen in Figure 11A.

In contrast, *MFN2* expression was not significantly affected by 7-KC (treatment factor: *p* = 0.5578; cell lineage factor: *p* = 0.2118; interaction: *p* = 0.1812), as seen in Figure 11B.

For *OPA1*, 7-KC induced marked and lineage-dependent changes (treatment factor: *p* < 0.0001; cell lineage factor: *p* < 0.0001; interaction: *p* < 0.0001). In AML-MSCs, 30 µM 7-KC increased OPA1 expression by approximately 5-fold compared with basal control (*p* < 0.0001), while 50 µM and 70 µM 7-KC produced more moderate increases of about 3.25-fold (*p* = 0.0184) and 2-fold (*p* < 0.0001), respectively. This strong induction at 30 µM was not observed in HD-MSCs (*p* < 0.0001 for AML-MSCs vs. HD-MSCs at 30 µM), whereas for 50 µM and 70 µM 7-KC, the higher OPA1 levels in AML-MSCs did not reach statistical significance compared with HD-MSCs (*p* = 0.5082 and *p* = 0.7373, respectively), as seen in Figure 11C.

For the fission gene *DRP1*, 7-KC significantly modified expression (treatment factor: *p* = 0.0054), with a borderline overall difference between lineages (cell lineage factor: *p* = 0.0528) and a significant treatment × lineage interaction (*p* = 0.0040). In AML-MSCs, DRP1 expression was increased at 30 µM 7-KC compared with basal control (≈4.5-fold, *p* = 0.0031) and was also higher than at 50 µM (*p* = 0.0428) and 70 µM (*p* = 0.0005). At 30 µM, DRP1 expression was significantly higher in AML-MSCs than in HD-MSCs, in which no change was detected (*p* = 0.0026), as seen in Figure 11D.

To assess the potential effects of 7-KC on mitochondrial electron transport chain components, we next evaluated *CYTB* and *ND1* expression.

For *CYTB*, two-way ANOVA did not show significant effects of treatment (*p* = 0.0751), cell lineage (*p* = 0.0670), or interaction (*p* = 0.1032), although a trend toward higher CYTB expression was observed in HD-MSCs compared with AML-MSCs at 30 µM 7-KC (*p* = 0.0523), as seen in Figure 11E.

In contrast, *ND1* expression was significantly reduced by 7-KC (treatment factor: *p* < 0.0001), with no effect of cell lineage (*p* = 0.7495) or treatment × lineage interaction (*p* = 0.9957), indicating a similar response in both HD-MSCs and AML-MSCs. Post hoc analysis showed decreased ND1 expression in HD-MSCs treated with 50 µM (*p* = 0.0021) and 70 µM (*p* = 0.0163) 7-KC compared with basal control, and in AML-MSCs treated with 30 µM (*p* = 0.0317), 50 µM (*p* = 0.0004) and 70 µM (*p* = 0.0197) 7-KC (Figure 11F).

Here, the transcriptional changes in mitochondrial dynamics and electron transport chain genes indicate that 7-KC imposes a coordinated, lineage-dependent remodeling of mitochondrial homeostasis in bone marrow MSCs. In the next section, we integrate these findings with the data on lipid transporters, nuclear receptors, Hedgehog signaling, stress/survival markers and redox balance to propose an overall model of 7-KC signaling in HD-MSCs and AML-MSCs.

### 2.6. Integrative Model of 7-KC Signaling in Bone Marrow MSCs

**Figure 12:**
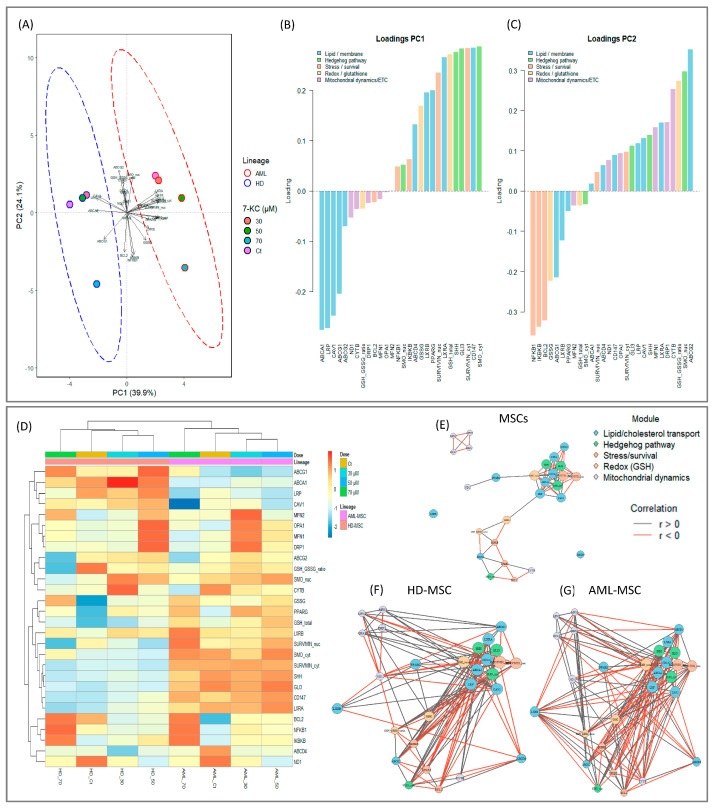
Integrative multivariate analysis of 7-KC signaling in bone marrow MSCs (*n* = 5 cell lines/group; 24 h). (**A**) PCA biplot (scores and loadings) built from lipid/oxysterol-handling markers (ABCs, lipid-sensing receptors, caveolin-1/LRP), Hedgehog components, stress–survival effectors, glutathione parameters, and mitochondrial dynamics/ETC genes. PC1 and PC2 explain 39.9% and 24.1% of the variance, respectively. Points represent experimental conditions and are colored by 7-KC concentration (Ct, 30, 50, 70 µM) and outlined by lineage (HD-MSC vs. AML-MSC); dashed ellipses indicate lineage clustering. (**B**,**C**) Variable loadings for PC1 (**B**) and PC2 (**C**), with bars colored by functional module (lipid/membrane, Hedgehog, stress/survival, redox/glutathione, mitochondrial dynamics/ETC). (**D**) Hierarchical clustering heatmap of z-scored variables across conditions, with top annotations indicating dose and lineage. (**E**) Correlation-based network for all MSC conditions (|r| ≥ 0.7), highlighting connectivity among lipid/cholesterol transport, Hedgehog, stress/survival, redox (GSH), and mitochondrial dynamics modules. (**F**,**G**) Lineage-stratified correlation networks inferred separately for HD-MSCs (**F**) and AML-MSCs (**G**). Node colors denote modules; edges represent correlations (black, r > 0; red, r < 0).

To integrate the multiple signaling and stress-related readouts modulated by 7-KC, we performed a principal component analysis (PCA) including ABC transporters, lipid-sensing receptors, Hedgehog pathway components, stress and survival markers, glutathione parameters, and mitochondrial dynamics/ETC genes. The first two principal components (PC1 and PC2) explained 39.9% and 24.1% of the total variance, respectively (cumulative variance: 64.0%), as seen in Figure 12A.

In the scores plot, HD-MSC conditions clustered on one side of PC1, whereas AML-MSC conditions clustered on the opposite side, indicating that PC1 primarily captured lineage-specific differences in the integrated response to 7-KC. PC1 was positively associated with cytoplasmic SMO, CD147, cytoplasmic and nuclear survivin, SHH, total GSH, LXRα/β and, to a lesser extent, GSSG, IKBKB and NF-κB, together with ABCD4. In contrast, negative loadings on PC1 were driven mainly by ABCA1, ABCG1, caveolin-1 and LRP, as well as ND1 and the GSH/GSSG ratio (Figure 12B).

Thus, positive PC1 scores (AML-MSC-like profiles) reflect a coordinated increase in Hedgehog-related features (SHH/SMO/GLI3), CD147–survivin–NF-κB-linked stress/survival signaling, an LXR/PPARγ-associated lipid-sensing program, and an expanded glutathione pool, whereas negative PC1 scores (HD-MSC-like profiles) are associated with a higher expression of cholesterol efflux and caveolae-related markers (ABCA1/ABCG1, caveolin-1, LRP), preserved ND1 expression, and a more reduced glutathione redox balance (Figure 12B).

PC2 captured an additional orthogonal gradient related mainly to stress signaling versus Hedgehog/mitochondrial features. The strongest absolute loadings on PC2 were observed for NF-κB, ABCG2, IKBKB and BCL2 (negative loadings), opposed by nuclear SMO, the GSH/GSSG ratio, CYTB, GSSG, DRP1, MFN1 and LXRα (positive loadings; Figure 12C). This axis therefore separates conditions in which NF-κB/IKBKB–BCL2 signaling is more prominent from those characterized by increased nuclear SMO, mitochondrial remodeling (DRP1/MFN1/OPA1), and a more oxidized or redistributed glutathione pool, Figure 12C.

A hierarchical clustering heatmap of z-scored variables provided a complementary view of these relationships. At the level of conditions, HD-MSC samples clustered together and were clearly separated from AML-MSC samples, while within each lineage the 7-KC-treated groups were ordered approximately according to dose, consistent with the PCA (Figure 12D).

At the level of markers, Figure 12D revealed three major clusters:

(i) A cholesterol efflux/caveolar module comprising ABCA1, ABCG1, caveolin-1, and LRP, with higher z-scored values predominantly in HD-MSCs;
(ii) A stress/survival–redox module including CD147, cytoplasmic and nuclear survivin, NF-κB, IKBKB, BCL2, total GSH, and GSSG, enriched in AML-MSCs and also increasing in HD-MSCs at higher 7-KC doses;
(iii) A Hedgehog/mitochondrial remodeling module comprising SHH, cytoplasmic and nuclear SMO, GLI3, MFN1, OPA1, DRP1, CYTB, and ND1, in which 7-KC exposure was associated with increased Hedgehog-related features and the remodeling of fusion–fission genes, accompanied by a trend toward reduced ND1 across treated conditions.

Correlation-based network analysis (|r| ≥ 0.7) further supported this integrative architecture, a tightly connected domains linking lipid/cholesterol handling (ABCA1, ABCG1, LRP, caveolin-1), Hedgehog components (SHH, SMO, GLI3), and stress–survival markers (NF-κB, IKBKB, BCL2, cytoplasmic/nuclear survivin), which in turn connected to redox and mitochondrial dynamics nodes (GSH total, GSSG, MFN1/2, OPA1, DRP1, ND1), as seen in Figure 12E.

When networks were inferred separately for HD-MSCs (Figure 12F) and AML-MSCs (Figure 12G), AML-MSCs displayed a denser stress/survival module coupled to lipid and Hedgehog nodes, consistent with more robust pro-survival rewiring under 7-KC exposure.

Here, the multivariate analyses support an integrated view of 7-KC signaling in bone marrow MSCs. The PCA indicated that the first two components are largely driven by coordinated changes in lipid/oxysterol handling (LXR/PPARγ, ABCA1/ABCG1, LRP, caveolin-1), Hedgehog markers (SHH/SMO/GLI3), stress–survival effectors (CD147, survivin, NF-κB, IKBKB, BCL2), and redox–mitochondrial readouts (GSH/GSSG, MFN1/2, OPA1, DRP1, and ND1), clearly separating HD-MSC and AML-MSC responses to 7-KC.

The hierarchical heatmap further revealed treatment-dependent clustering, with HD-MSCs exposed to higher 7-KC concentrations displaying a more oxidized, mitochondrial-sensitive profile, whereas AML-MSCs maintained a pattern enriched in stress and survival markers. Finally, the correlation networks highlighted a central nexus in which lipid receptors/transporters and Hedgehog components are tightly connected to stress–survival, glutathione, and mitochondrial modules, with a denser pro-survival wiring in AML-MSCs.

## 3. Discussion

In this study, we investigated how the oxysterol 7-KC reconfigures signaling networks in bone marrow mesenchymal stromal cells from healthy donors (HD-MSCs) and acute myeloid leukemia patients (AML-MSCs). By integrating readouts of lipid transporters and lipid-sensing receptors; morphogenetic pathway components; stress–survival axes; redox parameters; and mitochondrial metrics, our data indicate that 7-KC elicits a multimodal, lineage-dependent response. This response links cholesterol/oxysterol homeostasis to Hedgehog signaling, inflammatory and anti-apoptotic markers, and mitochondrial remodeling. We interpret these findings in the context of multidrug resistance (MDR) and leukemic niche biology, with an emphasis on ABC transporters and the LRP.

MDR in AML is often attributed to the expression of ABC transporters (ABCB1/P-gp, ABCCs/MRPs, and ABCG2/BCRP) and to additional determinants such as LRP. Collectively, these molecules have been described as modulators of intracellular trafficking and compartmentalization and have been linked to therapeutic response and relapse across different settings [12,20,56,57,58,59,60,61,62,63,64,65,66,67,68,69,70,71,72,73,74,75]. Nevertheless, much of the literature emphasizes mechanisms intrinsic to leukemic blasts; comparatively fewer studies have systematically examined how the bone marrow stroma contributes to a permissive, pro-MDR microenvironment.

Our results suggest that AML-MSCs display a basal phenotype consistent with enhanced stress tolerance and support of the leukemic clone. Compared with HD-MSCs, AML-MSCs exhibit lower baseline levels of efflux/caveolae-associated markers (LRP and ABCA1) and reduced activity of the LXRβ axis. Conversely, they show a stronger SHH/SMO/GLI3, CD147, and survivin signature, along with altered glutathione parameters, particularly GSSG. Taken together, these findings point to a preconditioned state in which adaptive and pro-survival signaling mechanisms are engaged even in the absence of treatment, consistent with a leukemic niche organized to promote cellular persistence and tolerance to stressors.

By demonstrating that 7-KC modulates transporters and components associated with membrane microdomains, as well as cell-survival markers, in MSCs, our study extends the MDR paradigm beyond the leukemic blast. In this scenario, MDR-associated proteins should not be viewed as exclusive to the leukemic clone; rather, they may represent part of a stromal signature that shapes oxidative stress handling, survival signaling, and potentially local pharmacodynamics and pharmacokinetics within the niche.

Given that 7-KC is an oxidized cholesterol derivative, we evaluated transporters involved in lipid/oxysterol efflux, with emphasis on the ABCA1/ABCG1 axis and other canonical LXR/RXR targets, which are key determinants of sterol efflux [76]; we also assessed ABCG2, a classic MDR-associated marker [77], in MSCs treated with subtoxic concentrations. Our data indicate that 7-KC promotes selective remodeling of the ABCA1/ABCG1 axis in HD-MSCs, whereas AML-MSCs display reduced plasticity, maintaining low ABCA1 levels and showing more discrete changes in ABCG1. Under our experimental conditions, we did not detect ABCC1 or ABCC2, and we observed only modest modulation of ABCG2. Together, these findings suggest that, in bone marrow stromal cells, the impact of 7-KC is more closely related to lipid homeostasis and stress adaptation than to a broad induction of canonical xenobiotic efflux pumps.

This pattern is consistent with the role of ABCA1 and ABCG1 in maintaining membrane composition, promoting sterol efflux, and decreasing lipotoxicity [78,79,80,81,82]. In HD-MSCs, 7-KC-induced upregulation of ABCA1 may reflect an adaptive response aimed at limiting sterol/oxysterol-driven lipotoxicity. In contrast, the blunted response in AML-MSCs suggests that the efflux machinery is already remodeled and less responsive. This may favor the persistence of lipid stress and contribute to maintaining a pro-survival state within the leukemic niche.

Evaluating nuclear receptors adds an important interpretive layer to our findings. Overall, 7-KC exerted only a modest acute effect on LXRα/β and PPARγ levels after 24 h, indicating that lineage-dependent differences are not primarily explained by changes in receptor abundance. Instead, the differential response may involve downstream functional regulation, including distinct target-gene and transporter signatures, as well as the reorganization of signaling pathways and their integration with other lipid environment-sensing circuits. This interpretation is consistent with evidence from other models showing that 7-KC can modulate LXR target genes, such as ABCA1 and ABCG1, while also eliciting context-dependent pro-inflammatory responses [83,84,85].

Surprisingly, we observed a nuclear pattern of ABCD4 in MSCs, in contrast to the predominantly cytoplasmic localization reported in control cell lines. Although the functional significance of this nuclear distribution remains uncertain, it raises hypotheses beyond the canonical role of ABCD4 in endomembrane compartments [86]. These include stress-associated relocalization processes and potential interfaces with lipid trafficking within nuclear compartments [87,88,89].

LRP and caveolin-1 complete the panel of membrane microdomain-associated proteins evaluated in this study. LRP is the main structural component of vault particles, large ribonucleoprotein complexes implicated in nucleo-cytoplasmic transport, intracellular compartmentalization, and multidrug resistance phenotypes, including effects on subcellular drug distribution and cellular stress adaptation [24,25]. Consistent with these functions, vaults are predominantly cytoplasmic, often enriched near the nuclear envelope and nuclear pore complexes. Their mobility is partly dependent on the cytoskeleton, supporting a role in intracellular trafficking [90]. In our study, LRP exhibited a largely perinuclear localization, with baseline differences between lineages and only modest modulation by 7-KC. Collectively, these observations suggest subtle changes in compartmentalization and trafficking under sterol-induced stress.

Caveolin-1 remained relatively stable, suggesting that 7-KC does not drastically disrupt caveolae. Instead, 7-KC may exploit these microdomains sites of convergence where lipid transporters, nuclear lipid sensors, and morphogen receptors converge. Caveolae are cholesterol- and sphingolipid-rich plasma membrane invaginations whose biogenesis and stability depend on caveolins; they function as metabolic and signaling domains, coupling membrane organization to signal transduction [91,92]. In MSCs, cholesterol–caveolin-1–caveolae homeostasis influences membrane properties and adhesion-related processes, underscoring the functional relevance of these microdomains in this cell type [93]. Moreover, direct evidence supports an interaction between caveolin-1 and 7-ketocholesterol, lending plausibility to the notion that 7-KC can modulate caveolar microdomains without necessarily collapsing caveolae structure [33]. Accordingly, our findings are consistent with the possibility that AML-MSCs maintain a constitutively remodeled oxysterol-regulation machinery, upon which 7-KC superimposes additional signaling cues, rather than acting merely as a nonspecific lipotoxic compound.

Hedgehog signaling is a key regulator of stemness, tissue repair, and leukemic niche function [42,94,95,96,97,98,99,100]. In our study, 7-KC modulated components of the SHH/SMO axis in a lineage-dependent manner. HD-MSCs responded to 7-KC with increased cytoplasmic and nuclear SMO. In contrast, AML-MSCs, already characterized by a stronger basal SHH/SMO/GLI3 signature, showed more limited additional changes. Total GLI3 levels did not vary markedly, suggesting that 7-KC may influence Hedgehog signaling primarily by affecting SMO subcellular localization, trafficking, and processing, rather than by inducing large changes in overall GLI3 abundance.

Oxysterols are recognized modulators of SMO, either through interactions with sterol-sensitive domains or indirectly by altering membrane cholesterol accessibility and the architecture of the primary cilium [40,41,43,101,102]. Our findings are consistent with a model in which 7-KC influences SHH/SMO signaling in MSCs, particularly in HD-MSCs. This adds a niche-related dimension and suggests the engagement of lipid stress adaptation programs. In AML-MSCs, the pathway appears to operate at a higher basal level, with a reduced dynamic range for further modulation.

A central finding of this study is that, beyond its pro-apoptotic effects, 7-KC also engages stress and survival pathways, particularly in AML-MSCs. CD147 is elevated in AML-MSCs under basal conditions and remains high after 7-KC treatment. This marker has been linked to extracellular matrix remodeling, invasion, and chemoresistance, and it has been implicated in the regulation of transporters such as ABCG2 across different tumor models [103,104]. Survivin showed a distinct pattern: It increased in the cytoplasmic compartment of HD-MSCs at higher 7-KC concentrations, whereas AML-MSCs maintained elevated basal levels, consistent with a pro-survival phenotype [44,105,106].

At the transcriptional level, 7-KC increased NF-κB, IKBKB, and BCL2, with more pronounced effects in AML-MSCs at the highest concentration tested. NF-κB is a central mediator of inflammatory and oxidative stress responses; IKKβ is a key kinase that drives NF-κB activation; and BCL2 supports mitochondrial integrity and resistance to apoptosis. Together, high CD147 expression and the upregulation of NF-κB/IKBKB, BCL2, and survivin in AML-MSCs delineate a stress-adapted cellular signature that may be more tolerant to 7-KC-induced lipotoxicity and, potentially, more likely to support a microenvironment permissive to therapy tolerance [46,107,108,109,110].

7-KC is frequently described as a prototypical oxysterol capable of inducing oxidative stress, mitochondrial dysfunction, and the oxiapoptophagy phenotype, characterized by the convergence of ROS production, apoptosis, and autophagy [50,51,111,112]. In our study, 7-KC induced distinct redox remodeling in HD-MSCs and AML-MSCs. In AML-MSCs, 7-KC primarily increased total GSH, expanding the glutathione pool and suggesting reinforced buffering capacity. By contrast, HD-MSCs exposed to higher concentrations showed a more pronounced increase in GSSG and a decrease in the GSH/GSSG ratio, consistent with an alteration toward a more oxidized state. Together, these patterns suggest that AML-MSCs may better control 7-KC-induced oxidative stress through glutathione-dependent mechanisms, whereas HD-MSCs tend to accumulate greater redox imbalance at higher 7-KC doses. Accordingly, we next assessed the impact of 7-KC on mitochondrial parameters.

Regarding mitochondrial function, 7-KC modulated mitochondrial membrane potential in a dose- and lineage-dependent manner. In HD-MSCs, TMRE fluorescence increased at intermediate concentrations (10–50 µM), consistent with mitochondrial hyperpolarization, and polarization was largely maintained at 100 µM. In contrast, AML-MSCs showed a pronounced reduction in the TMRE signal at 100 µM, indicating mitochondrial depolarization under the strongest challenge. Together, these findings are consistent with an early compensatory response in HD-MSCs at intermediate doses, whereas AML-MSCs exhibit reduced capacity to maintain ΔΨm at high 7-KC exposure. In parallel, under subtoxic 7-KC concentrations, ND1 expression was consistently reduced, whereas CYTB remained comparatively stable. This pattern suggests selective vulnerability of specific respiratory-chain components encoded by the mitochondrial genome, particularly under heightened oxidative and bioenergetic stress.

Mitochondrial dynamics were also reshaped by 7-KC, with lineage- and concentration-dependent modulation of fusion–fission genes, including MFN1, MFN2, OPA1, and DRP1. MFN1 and DRP1 were modulated in both lineages, whereas OPA1 increased more prominently in AML-MSCs at intermediate concentrations, consistent with an adaptive program that helps preserve mitochondrial network integrity and connectivity under stress. Accordingly, in AML-MSCs, the combination of elevated TMRE at intermediate doses, changes in OPA1 and DRP1, and the maintenance or upregulation of pro-survival markers such as BCL2 is compatible with tolerance-oriented mitochondrial remodeling that can buffer oxysterol-induced damage [113,114]. In contrast, in HD-MSCs, the abrupt decrease in TMRE at 100 µM, together with the trend toward reduced ND1 expression, suggests that high-dose 7-KC exceeds mitochondrial adaptive capacity, promoting loss of membrane potential and greater bioenergetic vulnerability.

In summary, our findings indicate that 7-KC induces redox alterations accompanied by signatures of mitochondrial remodeling, reinforcing the concept that mitochondria act both as targets and as active integrators of the cellular response to 7-KC. Moreover, because glutathione metabolism participates in cellular detoxification networks and functionally intersects with stress and survival responses, 7-KC-driven glutathione reconfiguration may contribute to ROS tolerance and to the maintenance of a pro-survival microenvironment within the leukemic niche. This interpretation is further supported by our multivariate analyses.

The PCA demonstrated a clear separation between HD-MSCs and AML-MSCs, indicating that 7-KC reprograms integrated cellular markers in a lineage-dependent manner. PC1 primarily reflected a contrast between a lipid-regulation and membrane microdomain axis and a set of Hedgehog- and stress–survival-associated features, coupled to changes in glutathione parameters. PC2 captured an additional dimension dominated by stress–survival and redox–mitochondrial signatures, suggesting that the 7-KC response is organized into modules that are partially orthogonal to the lipid axis. Consistently, the heatmap and hierarchical clustering showed that conditions segregated mainly by lineage and, secondarily, by dose. Correlation networks further supported connectivity among lipid/caveolar, Hedgehog, and stress–survival/redox modules, with a higher density of pro-survival interactions in AML-MSCs.

Integrating these layers, a model emerges in which 7-KC functions as an integrative signal linking sterol homeostasis, morphogenetic signaling, and stress responses in bone marrow MSCs. At baseline, AML-MSCs exhibit a stress-adapted state consistent with a pro-survival niche. This state is characterized by a reduced efflux and microdomain signature, higher activity of Hedgehog and survival axes, and a more challenged redox profile compared with HD-MSCs.

Upon 7-KC exposure, HD-MSCs display greater plasticity of the efflux axis and reorganization of Hedgehog components, albeit at a higher oxidative and mitochondrial cost at high doses. In contrast, AML-MSCs preferentially channel the response toward stress-tolerance circuits (NF-κB, BCL2, and glutathione) and adaptive mitochondrial remodeling, with more limited engagement of efflux programs. Collectively, these findings position the efflux and microdomains–Hedgehog–stress with redox–mitochondrial axis as a key determinant of the 7-KC response and as a set of candidate targets to understand—and potentially modulate—stromal contributions to therapy tolerance and MDR within the leukemic niche.

Although this study yielded relevant findings, several limitations should be acknowledged. First, we assessed a single exposure time point (24 h), which captures acute and early responses but does not address longer-term remodeling. Second, most readouts relied on fluorescence-based measurements and gene expression, without direct functional assessments of mitochondrial respiration, specific reactive oxygen species, or transporter activity. We also did not perform mechanistic perturbation approaches, such as knockdown or knockout of ABCA1, SMO, NF-κB/IKKβ components, or BCL2. Third, the number of primary AML samples was limited, and interpatient heterogeneity may influence both the baseline MSC phenotype and the magnitude of the 7-KC response. In addition, although MSCs were derived from bone marrow, ex vivo adaptation and expansion under standard culture conditions can reconfigure their transcriptional and functional phenotype, which may not fully recapitulate the in situ bone marrow microenvironment and niche architecture.

Our AML-MSC cohort included samples spanning different FAB subtypes (M1, M3, and M4), a broad age range (25–65 years), and both sexes; however, the limited sample size precluded robust stratified analyses by subtype, age, or sex. Likewise, HD-MSC controls reflected donor-to-donor variability, spanning young adult ages (20–41 years) and both sexes. Bone marrow was collected from different anatomical sites (distal femur and/or proximal tibia), which may influence baseline stromal composition and MSC properties. Together, these sources of biological and technical heterogeneity may contribute to inter-sample variability and may limit direct extrapolation to a single reference in situ bone marrow niche state.

In this context, future studies should test whether pharmacological inhibition of SHH/SMO, NF-κB, or BCL2, as well as targeted modulation of glutathione metabolism and mitochondrial dynamics, alters 7-KC responses in AML-MSCs and reverses a primed, stress-tolerant niche state. In addition, AML blast–MSC coculture systems and in vivo models will be essential to determine how 7-KC-induced changes in MSCs influence leukemic cell survival and responses to chemotherapy. Finally, the validation of key pathways at the protein and functional levels, for example, NF-κB/IKKβ phosphorylation, SMO trafficking and/or localization, LRP function, and ABC transporter-mediated efflux, will be essential. Together with the integration of clinical outcome data, we may establish stromal 7-KC-responsive signatures as candidate biomarkers and therapeutic targets in AML.

## 4. Materials and Methods

### 4.1. Study Design, Ethics, and Cell Lines

This study was conducted at the Laboratory of Histocompatibility and Cellular Immunity (LIM-19), Heart Institute (InCor), Hospital das Clínicas, Faculty of Medicine, University of São Paulo (HC-FMUSP), within the research group “Lipids, Oxidation and Cell Biology”. The protocol was approved by the HC-FMUSP Ethics Committee (CAAE 30797820.4.0000.0068; approval 4.011.280). All participants provided written informed consent. A total of 10 bone marrow-derived mesenchymal stromal cell (MSC) lines previously characterized according to ISCT criteria were used [115].

The HD-MSC cohort included five orthopedic surgery donors (2 females, 3 males) with a median age of 37 years (range, 19–70 years). HD-MSCs were isolated from bone marrow aspirates obtained from donors undergoing corrective knee surgery. Although these individuals were orthopedic patients, they had no known hematologic disorder and showed no clinical evidence of hematologic malignancy or other blood-related disease; accordingly, they were considered clinically healthy for hematologic parameters and were included as the healthy donor (HD) control group. Bone marrow samples were collected intraoperatively from the distal femur, proximal tibia, or posterior iliac crest, depending on anatomical accessibility and the surgical procedure performed.

The AML-MSC cohort comprised five patients (4 males, 1 female) with a median age of 29 years (range, 25–65 years). AML-MSCs were obtained from bone marrow aspirates collected at the posterior iliac crest (posterior superior iliac spine) during routine clinical procedures performed as part of the diagnostic evaluation of patients with acute myeloid leukemia (AML), as seen in Table 1. In all cases, approximately 10–15 mL of bone marrow aspirate was harvested under sterile conditions for subsequent mononuclear cell isolation, MSC culture, and expansion.

### 4.2. MSC Thawing, Culture, and Expansion

The MSCs used in the study were obtained from bone marrow aspirates processed under aseptic conditions in a laminar flow hood. Mononuclear cells were isolated after 1:1 dilution in PBS (pH 7.2–7.4) followed by density-gradient separation using Ficoll-Paque PLUS (3:1) and centrifugation at 450× *g* for 30 min at room temperature without braking. The plasma–Ficoll interface was collected and subjected to three PBS washes (3:1), with centrifugation at 450× *g* for 10 min, to remove residual contaminants. Cells were then counted and viability was assessed by trypan blue exclusion using a Neubauer chamber; cells were seeded in growth medium when viability reached 90%.

For primary culture and expansion, cells were maintained in low-glucose DMEM supplemented with 20% fetal bovine serum (FBS), sodium bicarbonate, and penicillin (100 U/mL)/streptomycin (100 µg/mL), plated in 75 cm^2^ flasks, and incubated at 37 °C in a humidified atmosphere containing 5% CO_2_. After 72 h, non-adherent cells were removed and the medium was replaced. Adherent cells displaying fibroblast-like morphology were maintained until colony-forming units developed, and upon reaching approximately 80% confluence, they were detached with trypsin/versene (ATV) for serial passaging and expansion up to passage 5 (P5). A fraction of the expanded MSCs was then cryopreserved as stock (1 × 10^6^ cells per vial) in low-glucose DMEM containing 10% FBS and 10% DMSO, and stored at −80 °C in an ultralow freezer at passages P2–P3.

For the experimental procedures, cryopreserved vials at P2–P3 were rapidly thawed at 37 °C, and their contents were diluted dropwise in low-glucose DMEM supplemented with 20% FBS, sodium bicarbonate, and penicillin/streptomycin. Cells were then centrifuged (250× *g*, 6 min), resuspended, and seeded into culture flasks under standard conditions (37 °C, 5% CO_2_, humidified atmosphere). The medium was replaced after 24 h to remove residual DMSO and dead cells, and subsequently renewed every 3 days until 80–90% confluence was reached, at which point the cells were again detached with trypsin/versene for re-expansion. Thus, all assays were performed using MSCs between passages P4–P6, with viability ≥95% as determined by trypan blue exclusion.

In parallel, MSC identity and characterization were confirmed by flow cytometry using FITC/PE/PerCP-conjugated antibodies (CD29, CD44, CD90, and CD105, with absence of CD11b, CD34, CD45, and HLA-DR). Briefly, 1 × 10^6^ cells were incubated for 20 min in the dark, washed (300 × g/5 min/4 °C), and 10,000 events were acquired on a FACSCalibur (BD Biosciences, San Jose, CA, USA) cytometer and analyzed using BD CellQuest Pro software, version 5.1. Maintenance of the undifferentiated state was assessed by RT-PCR using RNA extracted with TRIzol, followed by DNase treatment, cDNA synthesis, and detection of Oct-4 and Nanog, with GUSB used as the endogenous control and visualization on 2% agarose gel. Finally, multipotency was demonstrated by osteogenic differentiation (21 days, Alizarin Red staining) and adipogenic differentiation (15 days, Oil Red O staining) under appropriate inductive conditions.

### 4.3. 7-Ketocholesterol Preparation and Cell Treatment

7-ketocholesterol (7-KC) was synthesized from cholesterol and purity was confirmed by GC–MS (~98%) [116,117]. A stock solution (10,000 μM) was prepared in absolute ethanol and stored at −20 °C for up to 30 days. For assays, 7-KC was diluted in culture medium and cells were exposed for 24 h. Dose–response experiments initially used 10–100 μM 7-KC to estimate cytotoxicity and define subtoxic concentrations; subsequent experiments employed sub-IC_50_ doses (typically 30, 50, and 70 μM) for mechanistic profiling.

### 4.4. Viability, Mitochondrial Membrane Potential (ΔΨm), Caspase-3/7 Activation, and F-Actin High-Content Imaging Assays

MSCs were seeded at 5 × 10^3^ cells/well in 96-well plates and allowed to adhere for 24 h before treatment. Plasma membrane integrity was assessed by adding Hoechst 33342 before the endpoint and propidium iodide (PI) immediately prior to image acquisition. Images were acquired using an ImageXpress Micro High-Content System from multiple sites per well, and PI-positive cells were quantified using MetaXpress software., version 2.0 (Molecular Devices, San Jose, CA, USA). Mitochondrial membrane potential (ΔΨm) was evaluated using tetramethylrhodamine ethyl ester (TMRE, 50 nM). Cells were incubated with TMRE and Hoechst 33342 for 40 min at 37 °C, and TMRE fluorescence intensity was quantified by high-content imaging. Caspase-3/7 activation was assessed using the CellEvent Caspase-3/7 Green Detection Reagent in combination with Hoechst 33342, followed by high-content image acquisition; results were expressed as the percentage of positive cells. F-actin organization was analyzed after fixation with 4% paraformaldehyde, permeabilization with 0.1% Triton X-100, and staining with phalloidin–Alexa Fluor 568 and Hoechst 33342. Images were then acquired and analyzed by high-content imaging.

### 4.5. Metabolic Activity and Cell Cycle

To support the selection of subtoxic doses and evaluate functional impact: (1) MTT assay: MSCs were seeded at 5 × 10^3^ cells/well (96-well, sextuplicates), treated with graded 7-KC concentrations for 24 h, incubated with MTT (0.5 mg/mL, 4 h), solubilized in DMSO, and read at 570 nm (reference 650 nm). And (2) cell-cycle profiling: Hoechst-based DNA content analysis was performed after 24 h treatment using the high-content platform and MetaXpress cell cycle module; results were reported as % cells in G0/G1, S, and G2/M.

### 4.6. RNA Isolation, DNase Treatment, cDNA Synthesis, and RT-qPCR

For gene expression, MSCs were plated in 6-well plates and treated for 24 h. Total RNA was extracted using TRIzol, followed by chloroform phase separation and isopropanol precipitation. RNA was quantified by NanoDrop and samples meeting purity criteria were DNase-treated. cDNA was synthesized using the High-Capacity cDNA Reverse Transcription Kit. RT-qPCR was performed on a 7500 Fast Real-Time PCR System. Stress/survival targets, *BCL2* (Hs00153350_m1), *NF-KB* (Hs00968440_m1), and *IKKB1* (Hs00233284_m1) were quantified using TaqMan assays, and normalized to β-glucuronidase. The SYBR Green RT-qPCR primer pairs used for mitochondrial/dynamics-related genes were as follows (5′–3′; final concentration of each primer in a 10 μL reaction): MFN1 (NM_033540.2) F: TCCGCCTTTAACTTCTCGGG and R: GCCATTATGCTAAGTCTCCGC (400 nM); MFN2 (NM_014874.3) F: AATCTGAGGCGACTGGTGAC and R: CTCCACCAGTCCTGACTTCAC (200 nM); OPA1 (NM_015560.2) F: AGCCCTTCCTAGTTCAGAAGA and R: TCTTCCGGAGAACCTGAGGTAA (600 nM); DRP1 (NM_012062.4) F: GGGAGTAAGCCCTGAACCAAT and R: CCTACAGGCACCTTGGTCAT (600 nM); MT-CYTB (NC_012920.1) F: ACCCCCTAGGAATCACCTCC and R: GCCTAGGAGGTCTGGTGAG (200 nM); and MT-ND1 (NC_012920.1) F: ATACCCATGGCCAACCTCCT and R: GGGCCTTTGCGTAGTTGTAT (200 nM). Reactions were run in duplicate with stringent Ct reproducibility, and relative expression was calculated using the 2^−ΔΔCt^ method [118].

### 4.7. ABC Transporter Profiling by qPCR Array

A targeted ABC transporter expression screen was performed using a TaqMan^®^ Array Human ABC Transporters panel, comparing untreated versus 7-KC-treated (25 µM) conditions. RT-qPCR was run under manufacturer-recommended cycling conditions, and expression changes were computed relative to matched controls.

### 4.8. Indirect Immunofluorescence for Protein Readouts

MSCs were seeded in 96-well plates, treated for 24 h, fixed with 4% paraformaldehyde, permeabilized (0.1% Triton X-100), blocked (5% BSA), and incubated overnight at 4 °C with primary antibodies against lipid transporters/sensors and signaling proteins using the following sources and dilutions: anti-ABCA1 (Novus Biologicals, Centennial, CO, USA, 1:200), anti-ABCC1 (Abcam, Cambridge, UK, 1:50), anti-ABCC2 (Abcam, 1:50), anti-ABCD4 (Novus Biologicals, 1:500), anti-ABCG1 (Novus Biologicals, 1:100), anti-ABCG2 (Novus Biologicals, 1:250), anti-LRP (Abcam, 1:100), anti-CD147 (Abcam, 1:100), anti-SHH (Abcam, 1:400), anti-SMO (Novus Biologicals, 1:100), anti-GLI3 (Novus Biologicals, 1:100), anti-LXRα (Abcam, 1:50), anti-LXRβ (Sigma-Aldrich, St. Louis, MO, USA, 1:200), anti-PPARγ (Novus Biologicals, 1:200), anti-caveolin-1 (Abcam, 1:500), and anti-survivin (Abcam, 1:500). After washing, cells were incubated with fluorophore-conjugated secondary antibodies for 90 min at room temperature: R-phycoerythrin (PE, 1:500) for ABCA1, ABCC1, ABCC2, ABCG1, ABCG2, LRP, GLI3, and LXRα, or Alexa Fluor 488 (1:500) for ABCD4, SHH, SMO, LXRβ, PPARγ, caveolin-1, and survivin. Nuclei were counterstained with Hoechst 33342, images were acquired on an ImageXpress high-content platform, and fluorescence intensity and compartmental readouts (as applicable) were quantified in MetaXpress.

### 4.9. Cellular Glutathione Quantification

Total glutathione and oxidized glutathione were quantified using the GSH/GSSG-Glo™ Assay (Promega) following manufacturer guidance with minor adaptations. MSCs were seeded in white 96-well plates, treated for 24 h, and lysed under conditions enabling selective measurement of total GSH or GSSG, followed by luciferin generation/detection and luminescence acquisition in a GloMax^®^ Discover plate reader. The GSH/GSSG ratio was calculated from corrected luminescence signals.

### 4.10. Statistical and Integrative Analyses

Data are presented as box-and-whisker plots, in which the center line indicates the median, the box represents the interquartile range (Q1–Q3), and the whiskers indicate the minimum and maximum values and represent independent experiments performed across primary HD-MSC and AML-MSC lines, with technical replicates as indicated for each assay, duplicate wells and multiple imaging fields per well for high-content readouts. For conventional comparisons, statistical analyses were primarily conducted in GraphPad Prism 8.0.1 (GraphPad Software, Boston, MA, USA). Two-way ANOVA was used to test the main effects of lineage (HD-MSC vs. AML-MSC) and 7-KC dose, as well as their interaction. A significant lineage × dose interaction indicates that the magnitude and/or direction of the 7-KC response differs between HD-MSCs and AML-MSCs; therefore, post hoc comparisons were performed within each lineage and/or across all conditions, together with Tukey’s test, with multiplicity-adjusted *p*-values. Statistical significance was set at *p* ≤ 0.05, and adjusted *p*-values from the post hoc procedures were used for multiple-comparison inference.

To obtain an integrated, systems-level view of 7-KC-driven remodeling across lipid-processing, Hedgehog, stress–survival, redox, and mitochondrial features, multivariate and network-based analyses were performed in R software, version 4.5.0 (R Foundation for Statistical Computing, Vienna, Austria), using RStudio IDE, version 2026.01.1+403 (Posit Software, PBC, Boston, MA, USA). All quantitative variables were normalized prior to integration by z-score transformation, centered and scaled to unit variance, to place readouts measured on different scales on a comparable metric. Principal component analysis (PCA) was conducted using the base stats framework, prcomp, centered and scaled input, and results were visualized as score and loading plots; PC contributions and variable cos^2^ metrics were extracted when applicable. Hierarchical clustering and heatmap visualization were generated from z-scored matrices using distance-based clustering, typically Euclidean distance, and agglomeration via complete linkage, with marker- and condition-level dendrograms used to evaluate treatment-dependent clustering within and across lineages. For correlation structure discovery, Pearson correlation matrices were computed using pairwise complete observations, and correlation-based networks were inferred by applying a predefined threshold (|r| ≥ 0.7) to define edges. Network construction and topology metrics, degree and/or connectivity and centrality as needed, were implemented using igraph, and network visualization was performed with ggraph and tidygraph-compatible workflows where appropriate, with edge attributes representing correlation sign and magnitude.

The R environment relied on standard and widely used packages for reproducible multivariate analysis and visualization, including tidyverse for data wrangling, ggplot2 for graphics, FactoMineR and factoextra for PCA utilities and visualization, pheatmap and/or ComplexHeatmap for heatmaps and clustering displays, igraph and ggraph for network inference and plotting, and auxiliary packages such as reshape2 and/or tidyr for matrix reshaping, dplyr for data manipulation, and RColorBrewer and/or scales for plot annotation and palettes. Integrative outputs including PCA, heatmaps, and correlation networks were interpreted jointly to identify coordinated modules and lineage-specific rewiring patterns under 7-KC exposure.

## 5. Conclusions

This study demonstrates that 7-KC acts as a systemic modulator of bone marrow MSCs by coordinately reshaping oxysterol-related pathways, Hedgehog signaling, stress–survival programs, redox homeostasis, and mitochondrial dynamics. These responses were strongly lineage-dependent. HD-MSCs exhibited greater plasticity of the efflux axis and increased nuclear SMO, but also showed enhanced oxidative vulnerability and mitochondrial depolarization at high 7-KC concentrations. In contrast, AML-MSCs displayed a basally primed phenotype and preferentially redirected the 7-KC response toward stress-tolerance pathways, survivin upregulation, glutathione pool expansion, and adaptive mitochondrial remodeling, thereby preserving relative mitochondrial function at intermediate concentrations.

Multivariate and correlation-network analyses further indicate that these pathways are organized within an interconnected signaling network, supporting the notion that 7-KC may contribute to the maintenance of a pro-survival, therapy-tolerant leukemic niche. Overall, our findings highlight cholesterol efflux transporters, SMO, NF-κB/BCL2-associated signaling, glutathione metabolism, mitochondrial dynamics, and LRP as key nodes for understanding stromal contributions to resistance and as potential targets for future strategies aimed at modulating the AML microenvironment.

## Figures and Tables

**Table 1:** Clinical, hematologic, immunophenotypic, cytogenetic, and molecular characteristics of AML patients used for AML-MSC isolation.

AML-MSC	Age	Sex	Peripheral Blood Findings at Diagnosis	Morphology (FAB)	Karyotype (ISCN)	Immunophenotype	Molecular Findings	WHO 2022 Classification
01	26	Male	Hb: 7.8 g/dL; WBC: 63 × 10^9^/L; Blasts: 96%; Platelets: 130 × 10^9^/L	AML without maturation (FAB M1)	46,XY[20]	86.2%: CD45++; CD34++; HLA-DR++; CD117++; CD38+; CD11b+; CD13++; CD33+/−; CD22+	No recurrent mutation/translocation detected	AML, NOS
02	29	Female	Hb: 6.8 g/dL; WBC: 5.6 × 10^9^/L; Blasts: 76%; Platelets: 5 × 10^9^/L	Acute promyelocytic leukemia (APL; FAB M3)	46,XX,t(15;17)(q24;q21)[14]/46,XX[3]	50%: CD45+; HLA-DR−; CD34−; CD38+; CD117+; CD13+; CD33+; CD64+; MPO+	PML::RARA	Acute promyelocytic leukemia with PML::RARA
03	65	Male	Hb: 10.6 g/dL; WBC: 66.6 × 10^9^/L; Blasts: 85%; Platelets: 14 × 10^9^/L	Acute myelomonocytic leukemia (FAB M4)	46,XY[20]	CD45+/++; HLA-DR+/−; CD34−; CD117−; CD11b++; CD11c+; CD13+; CD14+/−; CD33++; CD36+++; CD64++; MPO++; CD56++	NPM1 mutation; FLT3-ITD	AML with NPM1 mutation
04	35	Male	Hb: 6.3 g/dL; WBC: 22.6 × 10^9^/L; Blasts: 30%; Platelets: 437 × 10^9^/L	AML with maturation (FAB M2)	Complex karyotype including t(9;22)(q34;q11.2)	CD45+; CD38+; HLA-DR−; MPO+; CD7+; CD56+	BCR::ABL1 (p210)	AML with BCR::ABL1 (de novo)
05	25	Male	Hb: 13.0 g/dL; WBC: 1.0 × 10^9^/L; Blasts: 7%; Platelets: 111 × 10^9^/L	AML without maturation (FAB M1)	47,XY,i(7)(q10),+i(21)(q10) [9]/48,idem,+mar [2]/46,XY[9]	CD7+; HLA-DR+; CD34++; CD38++; CD117++; CD11b+; CD13+++; CD33+; MPO+++	No recurrent mutation/translocation detected	AML, NOS

Note: Peripheral blood blast percentages are shown; in case 05, bone marrow blast infiltration was >20%, confirming the diagnosis of AML. Abbreviations: AML, acute myeloid leukemia; APL, acute promyelocytic leukemia; Hb, hemoglobin; WBC, white blood cell count; MPO, myeloperoxidase; NOS, not otherwise specified; SP, peripheral blood; BM, bone marrow; age in years. Peripheral blood blast percentages are shown; in case 05, BM blasts were >20%, supporting the diagnosis of AML.

## Data Availability

The data supporting the findings of this study are available from the corresponding author upon reasonable request. Due to ethical and privacy restrictions involving human-derived samples, the data are not publicly available.

## Abbreviations

The following abbreviations are used in this manuscript:

Abbreviation	Definition
7-KC	7-ketocholesterol
AML	acute myeloid leukemia
HD	healthy donor
MSC(s)	mesenchymal stromal cell(s)
AML-MSC(s)	AML patient-derived mesenchymal stromal cell(s)
HD-MSC(s)	healthy donor-derived mesenchymal stromal cell(s)
MDR	multidrug resistance
ABC(s)	ATP-binding cassette (transporters)
ABCA1/ABCG1/ABCG2/ABCD4	ATP-binding cassette transporters A1/G1/G2/D4
P-gp	P-glycoprotein (ABCB1)
MRP(s)	multidrug resistance-associated protein(s) (ABCC family)
BCRP	breast cancer resistance protein (ABCG2)
LRP	lung resistance-related protein
LXRα/β	liver X receptor alpha/beta
RXR	retinoid X receptor
PPARγ	peroxisome proliferator-activated receptor gamma
SHH	Sonic Hedgehog
SMO	Smoothened
GLI3	GLI family zinc finger 3
CD147	cluster of differentiation 147 (BSG/basigin)
Survivin (BIRC5)	baculoviral IAP repeat-containing protein 5
NF-κB (NFKB1)	nuclear factor kappa B (subunit 1)
IKKβ (IKBKB)	IκB kinase beta
BCL2	B-cell lymphoma 2
GSH/GSSG	reduced/oxidized glutathione
ROS	reactive oxygen species
TMRE	tetramethylrhodamine ethyl ester
ND1/CYTB	mitochondrially encoded NADH dehydrogenase 1/cytochrome b
MFN1/2	mitofusin 1/2
OPA1	optic atrophy 1
DRP1	dynamin-related protein 1 (DNM1L)
ETC	electron transport chain
PCA	principal component analysis
PC1/PC2	principal component 1/2
ANOVA	analysis of variance
SEM	standard error of the mean
PI	propidium iodide
PBS/DPBS	phosphate-buffered saline/Dulbecco’s phosphate-buffered saline
BSA	bovine serum albumin
DMSO	dimethyl sulfoxide
DMEM	Dulbecco’s modified Eagle’s medium
FBS	fetal bovine serum
RT-qPCR	reverse transcription quantitative PCR
IF	immunofluorescence
HCS	High-Content Screening
